# Experimental Evolution of *Escherichia coli* Harboring an Ancient Translation Protein

**DOI:** 10.1007/s00239-017-9781-0

**Published:** 2017-02-23

**Authors:** Betül Kacar, Xueliang Ge, Suparna Sanyal, Eric A. Gaucher

**Affiliations:** 1 NASA Astrobiology Institute, Mountain View, CA 94035 USA; 2 Organismic and Evolutionary Biology, Harvard University, 26 Oxford Street, Cambridge, MA 02138 USA; 3 Department of Cell and Molecular Biology, Uppsala University, BMC, Box-596, 75124 Uppsala, Sweden; 4 School of Biology, Georgia Institute of Technology, 950 Atlantic Drive, Atlanta, GA 30332 USA; 5 Petit H. Parker Institute for Bioengineering and Bioscience, Georgia Institute of Technology, Atlanta, GA 30332 USA

## Abstract

The ability to design synthetic genes and engineer biological systems at the genome scale opens new means by which to characterize phenotypic states and the responses of biological systems to perturbations. One emerging method involves inserting artificial genes into bacterial genomes and examining how the genome and its new genes adapt to each other. Here we report the development and implementation of a modified approach to this method, in which phylogenetically inferred genes are inserted into a microbial genome, and laboratory evolution is then used to examine the adaptive potential of the resulting hybrid genome. Specifically, we engineered an approximately 700-million-year-old inferred ancestral variant of *tufB*, an essential gene encoding elongation factor Tu, and inserted it in a modern *Escherichia coli* genome in place of the native *tufB* gene. While the ancient homolog was not lethal to the cell, it did cause a twofold decrease in organismal fitness, mainly due to reduced protein dosage. We subsequently evolved replicate hybrid bacterial populations for 2000 generations in the laboratory and examined the adaptive response via fitness assays, whole genome sequencing, proteomics, and biochemical assays. Hybrid lineages exhibit a general adaptive strategy in which the fitness cost of the ancient gene was ameliorated in part by upregulation of protein production. Our results suggest that an ancient–modern recombinant method may pave the way for the synthesis of organisms that exhibit ancient phenotypes, and that laboratory evolution of these organisms may prove useful in elucidating insights into historical adaptive processes.

**Electronic supplementary material:**

The online version of this article (doi:10.1007/s00239-017-9781-0) contains supplementary material, which is available to authorized users.

## Background

Understanding historical evolutionary pathways is crucial to understanding how life became the way it is today across millions of years of environmental and ecosystem change (Gould 1989). One of the most difficult aspects of characterizing these historical pathways is the limited amount of knowledge available about how ancient organisms behaved and changed through time. Fossils provide useful morphological and anatomical details, but only traces of information about sub-organismal processes and states can be inferred from fossilized specimens alone (Pagel 1999). Ancestral sequence reconstruction may provide a means of addressing this limitation of the fossil record; the technique permits phylogenetics-based sequence inferences of ancestral genes at the interior nodes of a tree using likelihood or Bayesian statistics and offers an opportunity to determine the selectively advantageous amino acid replacements responsible for changes in protein behavior associated with adaptive events for particular molecular systems (Benner 1995; Chang et al. 2002; Huelsenbeck and Bollback 2001; Liberles 2007; Pauling and Zuckerkandl 1963; Thornton 2004; Ugalde et al. 2004). Mathematical sequence reconstructions of ancient genes and their subsequent in vitro biochemical characterization alone, however, may not necessarily provide the salient details of why the protein evolved along a particular evolutionary pathway (Bar-Rogovsky et al. 2015; Copley 2012; Dean and Thornton 2007; Kacar 2016; Zhu et al. 2005). Incorporating a functional perspective into the study of ancient proteins was suggested to be instrumental for understanding historical adaptive pathways as well as bridging the evolution of protein-level function and the organism-level behavior, thus enabling predictions that connect inferred genotype to ancestral phenotype (Dean and Thornton 2007; Harms and Thornton 2013; Kacar and Gaucher 2013, 2012; Lunzer et al. 2005; Zhu et al. 2005).

**Fig. 1:**
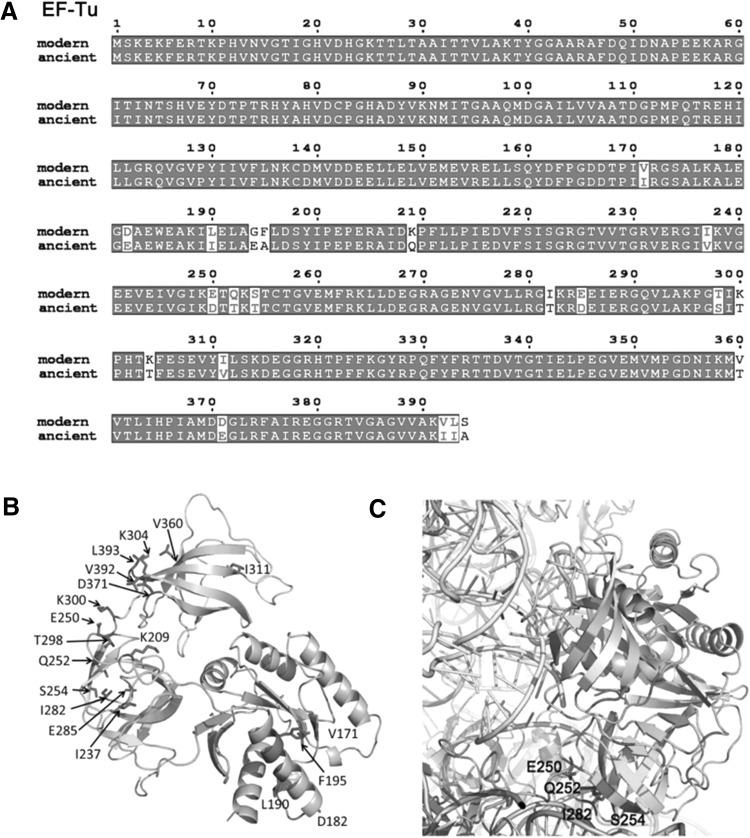
Sequence and structure analysis of EF-Tu. **a** Alignment of amino acid sequences of modern (AAC76364) and ancient EF-Tu from *E. coli*. Amino acid sequences were obtained from the NCBI database and aligned using Clustal Omega (Sievers et al. 2011). Figures were generated with the ESPript 3.0 server (Robert and Gouet 2014). Strictly conserved residues are shown in white. Partially conserved amino acids are boxed. Residues conserved in most of the members of one family are in *red font*. **b** The ribbon illustration of the EF-Tu adopted from the cryo-EM structure of *E. coli* ribosome–EF-Tu complex (PDB 5AFI). Domains I, II, and III are colored in slate, cyan, and wheat, respectively. The residues different in the ancient variant are shown with side chain (in *red*) and labeled accordingly. **c** Structure of EF-Tu–tRNA bound to the 70S ribosome in *gray* (PDB 5AFI) (Fischer et al. 2015) showing that the residues E250, Q252, S254, and I282 in domain II of EF-Tu were involved in the interaction with 70S ribosome. (Color figure online)

Previously, we proposed an evolutionary bioengineering approach to characterize the adaptation of an ancient protein to a modern genome on time scales of laboratory observation (Kacar and Gaucher 2012) (Fig. 1). This method builds upon heterologous gene replacement in bacteria, whereby the bacterial genome is introduced with a synthetic ancient gene. It remains to be seen, however, whether it is possible to elucidate and discern ancient adaptive steps from adjustments taken by a modern cell to a maladapted gene. When challenged with an ancestral component, will the engineered bacteria accumulate direct mutations on the ancestral component and “re-trace” the evolutionary history of this component by changing its sequence to be closer to the modern variant (Lind et al. 2010; Pena et al. 2010)? Alternatively, are compensatory mutations non-directional due to the very large solution space, and therefore the organism may be expected to respond to the ancient perturbation through modifications and modulation outside of the ancestral gene-coding region (Larios-Sanz and Travisano 2009)? To what degree will the adaptive pathways of the modified organism recapitulate the organism’s evolutionary history and thus allow researchers to address the role of chance and necessity at the molecular level? The key to resolving these prior questions is, at least in part, to assess the degree to which our system tracks or differs from experimental systems that replace genomic components with homologs obtained from other extant organisms (Acevedo-Rocha et al. 2013; Agashe et al. 2013; Andersson and Hughes 2009; Pena et al. 2010; Urbanczyk et al. 2012).

Our system relies on an organism with a short generation time and a protein under strong selective constraints in the modern host but its ancestral genotype and phenotype, if genomically integrated, would cause the modern host to be less fit than a modern population hosting the modern form of the protein. *E. coli* and an essential protein family of the bacterial translation machinery, elongation factor Tu (EF-Tu), are ideal for this type of experiment. *E. coli* is an organism that grows quickly in the laboratory, utilizes a range of energy sources, can be stored frozen, and later can be re-animated to test ancestral versus evolved populations, and the genetics of the organism are well known and easy to manipulate (Blount 2015). Elongation factor Tu (bacteria)/elongation factor 1 A (archaea and eukaryota) is a GTPase family member involved in the protein translation system (Kavaliauskas et al. 2012). EF-Tu forms a complex with GTP that in turn favors the binding of an aminoacyl-tRNA complex (Agirrezabala and Frank 2009). This ternary complex binds to mRNA-programmed ribosomes, thereby delivering aminoacyl-tRNA to the ribosomal A site (Czworkowski and Moore 1996). The biochemistry of EF-Tu has been studied for over three decades giving rise to a clear understanding of the functional aspects of the protein (Negrutskii and El’skaya 1998).

The reconstructed ancient EF-Tu protein represents that of an ancestral γ-proteobacterium that is inferred to be approximately 700 million years old, estimated based on molecular clock dating (Battistuzzi et al. 2004; Gaucher et al. 2003), and has 21 (out of 392) amino acid differences with the modern EF-Tu. Sequence and structure analyses suggest that ancient EF-Tu and modern EF-Tu exhibit similar properties (Fig. 1). Furthermore, the ancient EF-Tu protein exhibits the closest phenotypic property to the endogenous EF-Tu in terms of observed melting temperature (Tm) and its activity in a reconstructed in vitro translation machinery in which all other components necessary for translation besides EF-Tu are provided from the contemporary translation machinery (Gaucher et al. 2008; Zhou et al. 2012). This suggests that co-evolution between EF-Tu and aa-tRNAs/ribosome/nucleotide-exchange-factors in *E. coli* since the divergence of the ancestral and modern EF-Tu forms has not prevented the ancestral EF-Tu from interacting with the modern *E. coli* translation components (Kacar and Gaucher 2012).

*E. coli* bacteria have a paralogous copy of the EF-Tu gene *tufA*, in the form of *tufB*, that frequently recombines with the original copy (Abdulkarim and Hughes 1996). Each of the EF-Tu genes has its own specific expression machinery, and EF-Tu produced through *tufB* accounts for one-third of the cellular EF-Tu as that produced by the *tufA* gene in bacteria (Van Delft et al. 1987; van der Meide et al. 1983; Zengel and Lindahl 1982). Through recombination-mediated engineering (recombineering), the *tufA* gene was deleted from the bacterial genome and the *tufB* copy of a laboratory strain of *E. coli* was replaced with an ancient EF-Tu variant under the control of the endogenous *tufB* promoter. Ancient–modern hybrid populations were then evolved in replicate lineages through daily propagation of bacterial cultures in minimal glucose media (Bell 2016; Dragosits and Mattanovich 2013; Elena and Lenski 2003).

Evolved populations were sampled for whole genome sequencing, followed by identification of the total number of genomic changes in each population relative to the founding strain and subsequent assessment of the change in adaptive response through fitness assays. We further investigated whether in vivo analyses into the functionality of ancestral components can be used to discern effects arising from the substituted gene when screened from adaptive responses taken by the host cell to the sub-adapted genetic component. Taken together, this work provides the first demonstration of an artificial ancient essential gene variant inside a bacterial genome and provides insights into the principles of using experimental evolution for exploring adaptation of artificial genes in modern organisms.

## Results

### Replacement of Modern EF-Tu with Ancient EF-Tu is Detrimental to *E. coli* Fitness

**Fig. 2:**
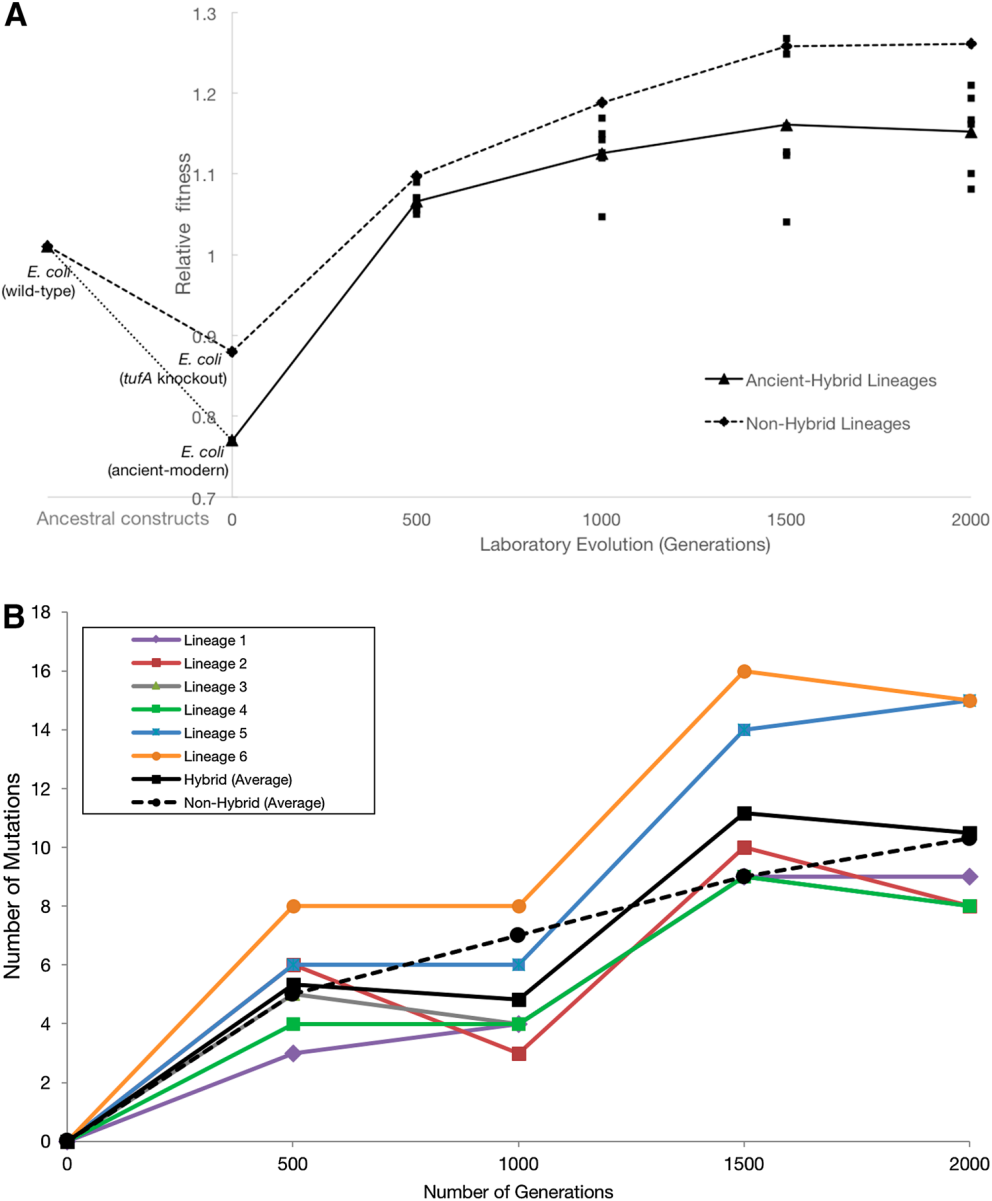
**a** Fitness values of *E. coli* populations relative to the ancestral strain during adaptive evolution. Replacement of the endogenous EF-Tu gene with the reconstructed ancient EF-Tu allele significantly reduces the fitness of the ancient–modern hybrid relative to the original strain (*dotted line*). Hybrid population mean fitness rapidly improved during experimental evolution in minimal glucose medium (*solid line*). *E. coli* ΔtufA represents the bacteria that contain a single tufB gene. Fitness change in E. coli ΔtufA is shown as *dashed line. Error bars* show 95% confidence interval among six replicate populations for each system. **b** Total number of mutations over time. Total number of genomic mutations accumulated in laboratory evolved hybrid and non-hybrid populations over time. Each *color* represents a sequenced hybrid genome. *Black lines* represent the average total number of genomic changes relative to the ancestor in each sampled hybrid (*solid*) and non-hybrid (*dashed*) lineages over time. (Color figure online)

Complete replacement of endogenous EF-Tu protein requires disruption of both native *tufA* and *tufB* genes and insertion of the inferred ancient gene (Supplementary Fig. 1) (Schnell et al. 2003). We first disrupted the native *tufA* gene. This intermediate *tufA* ^−^ *tufB* ^+^ construct displays a fitness of 0.89 (*P* < 0.001) relative to the parent clone. Subsequent replacement of *tufB* with the reconstructed ancient *tuf* gene produced a further fitness decline, to 0.77 (*P* < 0.001) relative to the parent clone (Fig. 2a). This dramatic fitness detriment of complete EF-Tu replacement suggests that the ancient gene is compatible with the modern *E. coli* genome, though unfit. Co-evolution between EF-Tu and aa-tRNAs/ribosome/nucleotide-exchange-factors in *E. coli* since the ancestral state for which the ancient tuf gene was inferred has thus not prevented the inferred ancestral EF-Tu from interacting with the modern *E. coli* translation system in a viable manner.

### Experimental Evolution Allows Bacteria to Restore Fitness

To examine the co-adaptation between *E. coli* and the ancient EF-Tu, we conducted evolution experiments with both the ancient–modern hybrid and the *tufA* ^−^ *tufB* ^+^ construct. Six replicate populations were generated for each of the two modified genomes by selecting identical clones that were verified to be free of any plasmid vectors that could mediate genetic exchange. The twelve populations were then evolved for 2000 generations under a daily 100-fold serial transfer regime in DM25 minimal glucose medium, at 37 °C and 150 rpm. Under these conditions, each population grew log_2_ (100) = 6.64 generations per day before reaching stationary phase. The daily maximum population size for each population is approximately 2.5 × 10^8^ cells. Fitness assays were conducted every 500 generations, in which evolved populations were competed against the ancestral clone. The ancient–modern hybrid populations displayed a mean fitness of 1.06 (*P* < 0.043) at generation 500 (Fig. 2a). This increased to 1.12 (*P* < 0.005) at generation 1000, and 1.15 (*P* < 0.009) and 1.16 (*P* < 0.001) at generations 1500 and 2000, respectively. The *tufA*− *tufB*+ populations also exhibited fitness increases. Mean fitness relative to the ancestor is 1.097 (*P* < 0.001) at generation 500, 1.16 (*P* < 0.004) at generation 1000, and 1.15 at both generations 1500 (*P* < 0.001) and 2000 (*P* < 0.004) (Fig. 2a).

### Comparative Analysis of Mutational Trends Across Lineages

In general, both the ancient–modern hybrid and non-hybrid control lineages acquired parallel responses at both the genetic and the physiological levels. All lineages exhibited comparable mutational rates over 2000 generations of laboratory evolution including a small fraction of synonymous mutations (Table 1, Supplementary Table 1). Non-synonymous mutations increased throughout the laboratory evolution trials on a per generation per lineage basis, a behavior observed in non-hybrid control lineages and in various adaptive evolution experiments (Barrick and Lenski 2013; Conrad et al. 2011; Herron and Doebeli 2013; Lang and Desai 2014; Tenaillon et al. 2016). Mutations were observed in the same suite of genes across all lineages, hybrid and control. Some of these mutations are reported from bacterial evolution experiments using minimal nutrient media (Table 1).

**Table 1:** Parallel mutations in genes for six, initially identical, independently evolved populations harboring an ancient EF-Tu

Gene	Function	Number of lineages that accumulated a non-synonymous mutation over 20% frequency				Present in non-hybrid lines?	Previously reported in evolution experiments?
		Generation					
		500	1000	1500	2000		
mreB	Cell wall structural complex MreBCD, actin-like component MreB	2	2	4	4	Yes	Yes
mrdB	Cell wall shape-determining protein	1	2	2	2	Yes	Yes
thrT/tuf	tRNA-Thr/protein chain elongation factor EF-Tu	2	3	5	5	No	No
fadA	Acetyl-CoA acteyltransferase	2	2	3	4	No	Yes
ftsZ	Cell division protein FtsZ	1	1	2	5	No	Yes
icIR	DNA-binding transcriptional repressor	-	1	4	3	No	Yes
accC	Acetyl-CoA carboxylase	-	1	3	5	Yes	Yes
pykF	Pyruvate kinase	-	1	3	5	Yes	Yes
topA	DNA topo isomerase	1	1	1	1	Yes	Yes
nusA	Transcription elongation factor NusA	1	1	1	1	No	Yes
infB	Translation initiation factor IF-2	1	1	1	-	No	Yes
hupA	HU, DNA-binding transcriptional regulator, alpha subunit	1	1	1	1	No	Yes

Top part represents the genes that accumulated mutations in at least three populations containing the ancient EF-Tu gene and occupied the population by minimum 20% across generations 500 to 2000 are shown for a total of six populations evolved in parallel. thrT/tuf represents the intergenic region between ancient EF-Tu gene and thrT gene

The bottom three are the mutated genes that are specific only to the single lineage that did not accumulate a mutation in the thrT/tuf region (Lineage 6).

Prior laboratory evolution studies that report mutations in genes that are also detected in our study include Barrick et al. 2009, Maddamsetti et al. 2015, Dillon et al. 2016, Conrad 2009, Herron and Doebeli 2013 and Phillips et al. 2016

We sought to answer whether the hybrid strains exhibited a unique response to the challenge of functioning with the (maladapted) engineered ancient EF-Tu protein. The genomic sequences of ancient–modern hybrid strains differed from the evolved non-hybrid lineages (*E. coli* strain lacking a *tufA* gene) in distinct ways. First, four single-nucleotide polymorphisms were located only in the hybrid lineages within the coding regions of three known genes, *fadA, ftzZ*, and *iclR*. Second, five out of six evolved ancient–modern hybrid lineages exhibited mutations in the intergenic region *thrT*/*tufB*, which corresponds to the promoter region of the engineered ancient EF-Tu gene (a 19-bp duplication and three other independent SNPs) (Fig. 2a). One population (Lineage 6) stood out as unusual as the *thrT*/*tufB* region in this lineage did not mutate. This lineage also exhibits the highest amount of non-synonymous mutations per generation (Fig. 1b; Supplementary Table 1).

### *Promoter-Level* Mutations Upregulate Ancient EF-Tu Expression and Restore Bacterial Fitness

To identify the genetic bases of the observed fitness increases, whole genomes of clones were periodically isolated and sequenced from all six evolved populations during the evolution experiment. Mutations generally accumulated in similar genes across all experimental construct populations (Table 1). However, five out of the six ancient–modern hybrid lineages (and none of the other control lineages) evolved mutations in the *thrT*/*tufB* promoter region, with four variant alleles being observed (Lee et al. 1981). The majority of these *thrT*/*tufB* promoter region mutations accumulated early in the experiment and rose to high frequency, if not fixation, by 2000 generations across all five populations in which they occurred (Fig. 2a, b). Such cis-regulatory mutations have been shown to be a common means of adaptation (Hoekstra and Coyne 2007; Jacob and Monod 1961; Lynch and Wagner 2008). By contrast, we observed no mutations in the ancient or modern EF-Tu gene-coding region in any of the evolved lineages, suggesting that compensatory amino acid replacements may have only occurred at other sites in the genome.

**Fig. 3:**
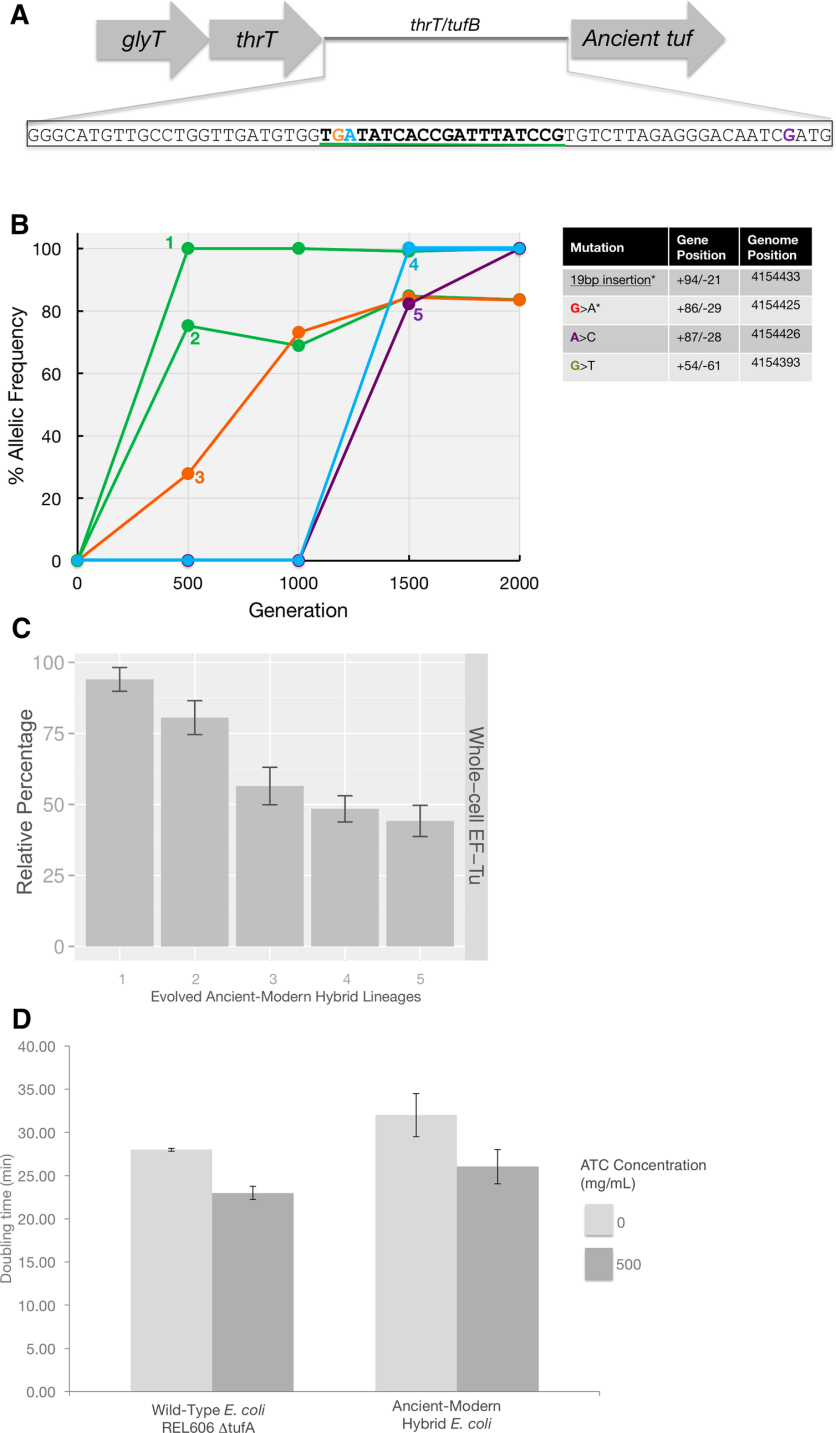
Analysis of the mutations accumulated in the cis-regulatory region thrT/tufB. **a** The thrT/tufB promoter region in which five of six evolved hybrid populations were found to have accumulated mutations. **b** Allelic frequency of the mutations located in ancient EF-Tu gene’s promoter region per generation per population during laboratory evolution. **c** Relative abundance of ancient EF-Tu protein among evolved hybrid strains using the peak area quantification from MS proteomics data. *Error bars* obtained using ANOVA/*t* test. **d** Growth rates of an isogenic strain of *E. coli* REL606 lacking the *tufA* gene, as well as the unevolved ancient–modern hybrid *E. coli*, were evaluated in the presence of Anhydrotetracycline (ATC) inducer. Strains were induced with 500 mg/mL ATC in rich growth media for 3–4 h to achieve proper induction. Cells from these fresh induced cultures were inoculated in 96-well plates and grown at 37 °C with a starting OD_600_ of ∼0.06 under respective ATC concentration. Doubling times were determined by fitting the exponential growth curves with an exponential function

We performed whole cell shotgun proteomic analysis on five of the evolved hybrid populations with EF-Tu promoter mutations to examine the impact of these mutations on EF-Tu protein levels. The assayed time points were those for each population at which the mutations had reached over 90% frequency in the population. For comparison, we also assayed unevolved ancient–modern hybrid bacteria, the wild-type parent *E. coli* strain, and an unevolved *tufA* ^−^ *tufB* ^+^ construct. Deletion of the *tufB* copy and the subsequent insertion of the ancient reconstructed gene into *E. coli* cause EF-Tu protein levels to drop by approximately ~66% relative to that observed in the wild type. The evolved hybrid populations with *tufB* promoter mutations all show significant increases in EF-Tu levels (Fig. 3c). We also assessed the effect of these promoter mutations on EF-Tu expression level in vitro by examining their effect on a plasmid-borne fluorescent reporter. The mutant promoters increase expression between 1.5- and 20-fold (Supplementary Fig. 2). Interestingly, promoter mutations that rose to high frequency later during the experiment had lower relative effects on ancient EF-Tu protein expression than those that did so earlier during laboratory evolution. To test whether increased ancient EF-Tu levels would correlate with increased fitness, the unevolved ancient–modern cells and *E. coli* harboring a single wild-type *tufB* gene were transformed with pASK plasmids expressing ancient EF-Tu proteins (Materials and Methods). Over-expression of ancient EF-Tu protein in *E. coli* isogenic strain in rich media decreases the doubling time from 26 to 22 min. Similarly, over-expression of ancient EF-Tu protein in ancient–modern hybrid ancestor decreases the doubling time from 33 to 26 min (Fig. 3d). This observation is in agreement with previous studies demonstrating the correlation between the cellular concentration of EF-Tu and organismal fitness (Brandis et al. 2016; Tubulekas and Hughes 1993). Taken together, these results indicate that each experimental population exhibited parallel patterns of response such as upregulation of EF-Tu, as well as more idiosyncratic means of compensating for altered EF-Tu expression and activity.

### Replacement of the Endogenous EF-Tu with the Ancient Counterpart Abolishes Previously Existing Protein-Level Interactions

**Fig. 4:**
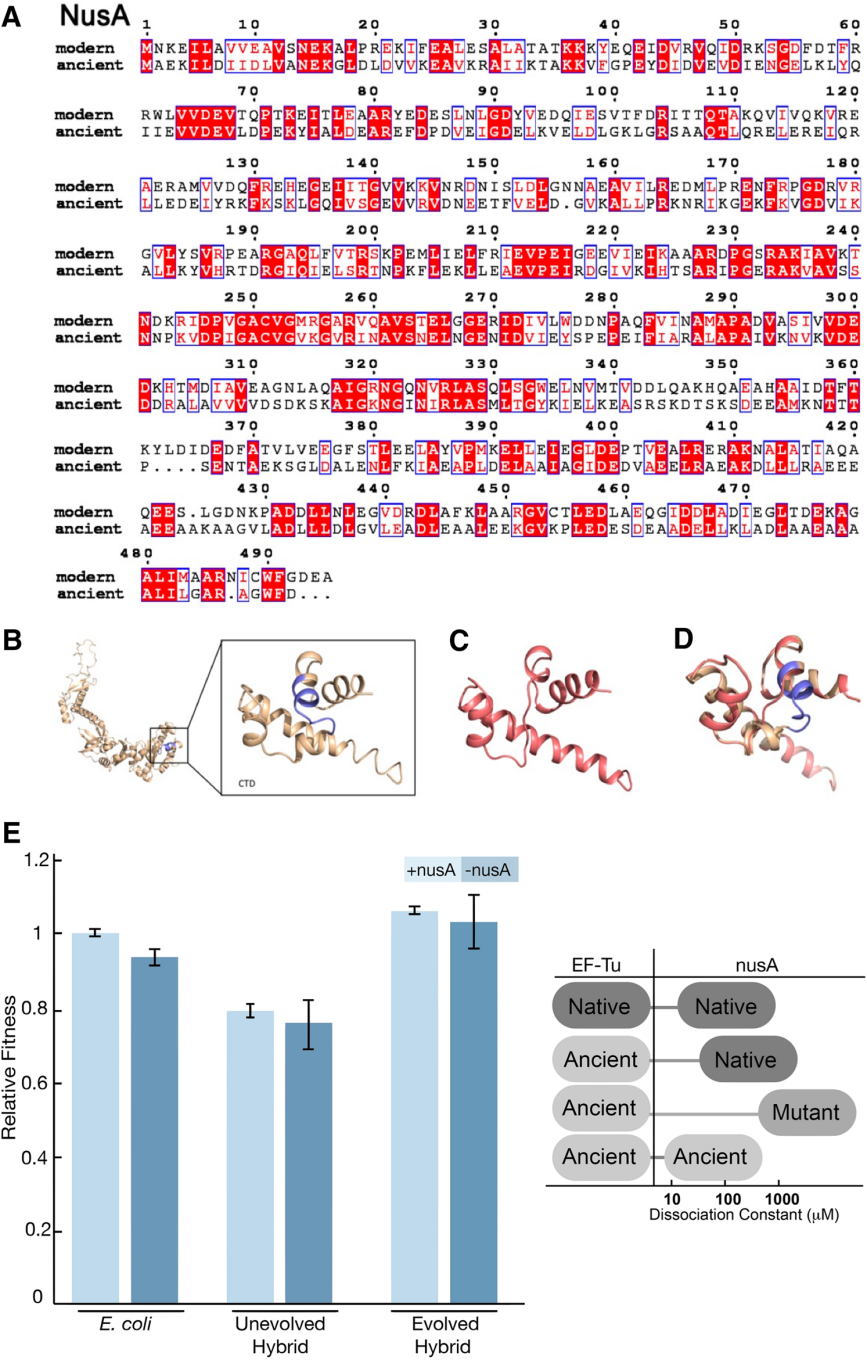
Sequence and structure analysis of NusA. **a** Alignment of amino acid sequences of modern and ancient NusA. Amino acid sequences were obtained from the NCBI database (AAC76203), the alignment and figure display were performed the same way as in Fig. 1. **b** Predicted model of *E. coli* NusA based on the crystal structure of NusA from *Planctomyces limnophilus* (4MTN) by the use of SWISS-MODEL (Biasini et al. 2014). The structure of the C-terminal domain demonstrates the deletion of nine residues (*colored slate*). **c** Structure prediction of the mutant NusA protein, harboring nine amino acid deletion in its C-terminal domain (CTD). **d** Wild-type NusA (colored wheat) and the mutant NusA with deletion of nine amino acids (colored salmon) alignment demonstrates the confirmational changes. **e** Fitness change after deletion of nusA gene from the ancestral and evolved bacterial genome. (*Left*) Bacterial constructs with isogenic nusA knockouts are generated and competed against the native *E. coli* bacteria for fitness measurement. (*Right*) The interactions between the native EF-Tu, ancient EF-Tu, and nusA variants are measured via in vitro isothermal calorimetry binding assays. (Color figure online)

There was one ancient–modern hybrid lineage that lacked the tufB promoter mutations and instead accumulated a number of unique mutations not found within the other lineages, including one in the *nusA* gene (Table 1). The *nusA* gene is a translation regulator and its protein product is thought to exhibit chaperone activity with direct interaction to ribosomal proteins (Shazand et al. 1993). Furthermore, the NusA protein has been suggested to be one of the earliest proteins, with a fundamental role in cellular translation machinery (Charlebois and Doolittle 2004) and that NusA may affect the efficiency of the translation machinery in a manner similar to EF-Tu and other ribosomal proteins with chaperone activity (Caldas et al. 2000, 1998). To examine the biochemical effects of a mutation acquired by the nusA gene during laboratory evolution (specifically, a 27-bp deletion of the NusA protein C-terminal domain), mutant *nusA* gene was cloned in an expression vector and subsequently purified (Fig. 4a). Changes in the interaction of the mutant NusA protein with EF-Tu, ancient or modern, were examined by measuring protein–protein binding via isothermal titration calorimetry. While the wild-type EF-Tu bound NusA with a robust binding constant (Kd) of 14.6 ± 5.2 µM, the ancient EF-Tu binds only weakly to the native NusA protein. Moreover, dipeptide formation assays detected no NusA-EF-Tu interaction in the ribosome, and the interaction between EF-Tu and NusA had no observable effect on dipeptide formation in the ribosome (Supplementary Fig. 4).

On the other hand, mutant NusA and ancient EF-Tu exhibit a Kd of 680 ± 66 µM, suggesting virtually no interaction (Supplementary Fig. 4). The loss of interaction might be due to the lack of interaction between EF-Tu in the ancestral context in which the reconstructed ancient EF-Tu existed. To test this hypothesis, ancient nusA gene representing the γ-proteobacterial ancestor was phylogenetically reconstructed, synthesized, expressed, and purified, and the ancient NusA protein’s capacity to bind to the ancient EF-Tu protein was examined (Fig. 4a, b). While the replacement of modern nusA with its ancestral counterpart decreases organismal fitness, deletion of the mutant nusA gene from the evolved and the ancestral ancient–hybrid allele has no observable fitness effect at the organism level. There is no detectable interaction between the two ancient counterparts of the two proteins (Supplementary Fig. 4). These results suggest that the nusA mutation is neutral in evolved hybrid background and the mutation occurred independently of the ancient EF-Tu protein mechanism.

### Discussion

By combining a unique set of tools drawn from synthetic biology, evolutionary biology, and genomics, we experimentally evolved and then analyzed the adaptive properties of a single-celled organism with a genome containing a reconstructed ancient gene. A majority of the evolved lineages accumulated mutations in the promoter region of the ancestral *tuf* gene and these lead to increased expression of the ancient EF-Tu protein. It is possible that these promoter mutations constitute the most facile pathway for compensatory genetic changes, particularly for highly conserved essential proteins. Structural mutations in the ancient *tuf* gene might have been observed had evolution continued and the capacity for response via these facile pathways exhausted. Although the artificial genetic sequences themselves were not subject to mutation in the course of laboratory evolution on the observed scales, it is nevertheless possible that the cellular and genetic adaptations to these synthetic genes recapitulate fundamental aspects of adaptive constraints that acted more broadly upon predecessors within the clade.

Understanding the lack of direct accumulation of mutations on the ancient EF-Tu requires a full accounting of the fitness effects of all potentially contributing mutations. Considering the important role of EF-Tu in the translational machinery, mutations accumulating directly on the EF-Tu gene can cause cell lethality and thus may not be readily adaptive (Goldman et al. 2010; Kacar and Gaucher 2013; Pereira-Leal et al. 2006). On the other hand, there are likely to be beneficial mutations that can occur without causing cell lethality, but they do not confer the advantage that others do under the same conditions, resulting in tuf mutations always being outcompeted within earlier generations. Increasing the cellular EF-Tu protein level may be the first response of the organism to survive with a drastic alteration introduced by a maladapted protein central to the translation process (Bridgham et al. 2009; Gong and Bloom 2014; Kryazhimskiy et al. 2014; Kvitek and Sherlock 2011; Lunzer et al. 2010).

Engineering native genomes with ancient genes has been considered a challenging experimental approach due to the possibility of functional incompatibility of the ancestral genes in modern organisms (Hobbs et al. 2015). Moreover, altering essential genes carries the risk of drastic effects on cellular epistatic networks (Coulomb et al. 2005; Drummond et al. 2005; Zotenko et al. 2008)—indeed, a single mutation in the translation machinery can drastically impact an organism’s viability (Ito et al. 1998; Lind and Andersson 2013). Ancestral sequences could be maladapted to the host cell to the extent that a functional organism is all but precluded due to phenotypical alteration introduced by subsequent functional divergence and promiscuity over time (Copley 2003), However, this experimental limitation does not apply to reconstructed ancestral genes alone. It has been suggested that as the number of nodes connecting an extant protein within its protein–protein interaction network increases, the capacity to replace that protein with another homolog decreases despite the presumed functional equivalence between the endogenous gene and the homolog (Jain et al. 1999). Several orthologous protein substitution experiments demonstrated successful transfer of an alien gene to a foreign genome (Bershtein et al. 2015; Larios-Sanz and Travisano 2009; Lind et al. 2010), resulting in a fitness decrease similar to our approach, which was referred to as the “weak link approach” (Counago et al. 2006). After evolving these organisms under laboratory conditions, it was demonstrated that direct accumulation of convergent mutations on the extant alien gene exhibits adaptive behavior (Counago et al. 2006; Miller et al. 2010; Pena et al. 2010). In this study, however, we detected that adaptive mutations compensating for the fitness detriment of the suboptimal ancestral gene occurred outside of the foreign gene that was introduced, including within the promoter region of the ancient gene.

Overall, the evolved hybrid populations exhibited similar behaviors—increased ancient protein levels in hybrid populations are coupled to increased fitness, mutations on similar genes in independently evolving lineages, and lack of adaptive mutation within the ancestral gene. It is possible that the most consistent drivers of historic mutational change may be macroscopic variables (i.e., atmospheric composition, nutrient availability, ecological partitioning, or long-term climate fluctuations) that are not typically incorporated into laboratory-scale synthetic evolution experiments. In just one example relevant to this experimental setup, the EF-Tu protein phenotype is tightly coupled to the optimal growth temperature of its host organism (Gromiha et al. 1999), but bacterial clades are not thought to have gone through any significant temperature-dependent evolutionary bottlenecks over the 700 million years of evolution that has occurred between the ages of the modern and ancestral homologous sequences (Blattler and Higgins 2014; Knauth 2004). This is one possible interpretation for the observed lack of mutations on EF-Tu itself which may form the basis of a testable hypothesis that specifically links cell-level response to a foreign gene to laboratory-controlled evolvability. The role of the environment (in this case, a glucose-limited minimal medium) in determining the co-adaptation specificity between ancient EF-Tu and *E. coli* bacteria in evolving populations should be studied in greater detail, such as monitoring the evolutionary pathway of the hybrid populations under a range of alternative environmental conditions.

### Conclusions

Engineering bacterial genomes with phylogenetically reconstructed genes complements the current technique of genome-level alterations of gene and gene clusters with currently existing homologs, and provides insights into molecular mechanisms of adaptation by providing access to the historical states of currently existing proteins. However, these methods are also severely constrained by limited existing knowledge of how laboratory evolution setups impact evolutionary trajectories. This knowledge is critical for discerning the change in behavior due to the ancestral state of the protein from the change in system-level behavior attributable to its intrinsic response to a suboptimal cellular component. The synthetic system described here may enable the development of ancient–modern hybrid model systems that will provide new insights related to the role of evolutionary history and the “tape” of evolution, as well as the degree of coupling between protein-level biochemical attributes and macroscale evolutionary trajectories and biogeochemical cycles. Relatedly, future evolutionary synthetic geobiology applications of this method could focus on linking substituted component behavior with a demonstrable organismal phenotype that can be independently retraced over the inferred age of the component (Kacar et al. 2016).

## Methods

### Bacterial Strains and Culture Conditions

All experiments were conducted at 37 °C unless stated otherwise. Luria–Bertani (LB) broth was used as the base medium for liquid cultures and agar plates. Experimental evolution and competition assays were carried out in Davis–Mingioli minimal medium (DM) supplemented with 25 mg/L glucose (Davis 1950). We used minimal glucose media to enable comparison with similar laboratory evolution experiments using REL606 strains. Tetrazolium arabinose (TA) plates were used as the base for competition experiment plating. When required, LB and DM media were supplemented with kanamycin, chloramphenicol, and tetracycline antibiotics. All dilutions were carried out in 0.1% sterile saline. LB and DM cultures were incubated on a rotary shaker at 200 and 150 rpm, respectively. The REL606 parental strain was kindly donated by Richard Lenski. DNA sequence encoding the ancestral EF-Tu protein was inferred on the basis of the genetic code, codon optimized for efficient expression in *E. coli*, chemically synthesized by DNA 2.0, and cloned into a pET15b plasmid as reported previously (Gaucher et al. 2008).

### Recombineering

#### Construction of the Ancient–Modern Hybrid Strain

Integration of the ancient EF-Tu gene (*AnEF*) into the chromosome of *E. coli* strain REL606 was carried out via the λ-red homology recombineering approach as described by Datsenko and Wanner (Datsenko and Wanner 2000). First, linear DNA containing homology sequences upstream and downstream of *tufA* gene was amplified by PCR, via 5′ GTGGTTGCGAAAATCATCGCTAGAATTCCGGGGATCCGTCGACC 3′ and 5′ TGTAATTAGCCCAGAACTTTAGCAACTGTAGGCTGGAGCTGCTTCG 3′, and pKD13 plasmid as a template, and then transferred in REL606 cells through electroporation, together with the temperature-sensitive pKD46 plasmid. Recombinants were isolated from LB agar plates containing 50 µg/µL kanamycin at 37 °C, grown in liquid LB medium containing 50 µg/µL kanamycin, and their genomic DNA was isolated using Promega Wizard Genomic DNA Purification Kit. Confirmation PCR was performed using genomic DNA isolated from colonies as a template, with primers aligning to the chromosome outside of the recombination site (5′ CAGGCCGTAATTGAAGCCCGTGGTAAATAAGCC 3′ and 5′ GAATAATTTATTCGTTCTGACAGTACGAATAAG 3′). Once the successful replacement of *tufA* gene with the kanamycin marker was confirmed via Sanger sequencing, the strain was transformed with linear DNA containing homology sequences upstream and downstream *tufB* flanked in between the AnEF DNA construct soed to a chloramphenicol marker originally amplified from the A007 loxP-Cm-loxP plasmid (Gene Bridges GmbH) via Gibson Assembly. The transformants were selected on LB plates containing 25 µg/µL chloramphenicol and 50 µg/µL kanamycin at 37 °C and the correct insert was screened with primers aligning to the chromosome outside the recombination site using Forward primer 5′ TCCGTGTCTTAGAGGGACAATCGATG 3′ and Reverse primer 5′ GCAATTAGCTCAGAACTTTTGCTAC 3′. Once confirmed, both the kanamycin and chloramphenicol markers were removed using pCP20 and 706-Cre plasmids (Gene Bridges GmbH), respectively, followed by the confirmation of the deletions by genomic PCR analysis. Plasmids pKD46 and pCP20 were cured by growing the cultures at 42 °C, and the final Δ*tufA*, Δt*ufB*:AnEF construct was moved into a fresh ancestral strain via bacteriophage P1 transduction. Freezer stocks of the REL606 Δ*tufA*, Δ*tufB:AnEF* were prepared by mixing 50% sterile glycerol and overnight liquid cultures originated from a single colony in 1:2 ratios. All stocks were stored at −80 °C. Isogenic Ara+ variants of the REL606 Δ*tufA*, Δ*tufB:AnEF* were obtained through gene-gorging protocol (Herring et al. 2003) (plasmid pJEB12 is kindly donated by Jeff Barrick).

#### Deletion of nusA Gene from the Chromosome

The nusA gene from the chromosome of REL606, ancestral REL606 Δ*tufA*, Δ*tufB:AnEF*, and evolved REL606 Δ*tufA*, ΔtufB:AnEF strain from lineage 2 were replaced with a FRT-kan-FRT fragment in the presence of pKD46 helper plasmid as described by Datsenko and Wanner using primers 5′ TCCTGCGTGAAGATATGCTG 3′ and 5′ TCACTTCTTCGCCGATTTCT 3′. PCR amplification of the recombination region and Sanger sequencing of this amplified region confirmed the correct replacement and the removal of the selection cassette. The cassette was then removed from the chromosome via pCP20, followed by the curation of pKD46 and pCP20 plasmids at 42 °C.

### Growth Assays

Saturated overnight cultures were preconditioned by dilution into sterile saline by a 1:100, then again by 1:100 into DM25 medium, followed by an overnight growth. Preconditioned cultures were diluted 1:100 into the assay medium and 100 µL was transferred into a 96-well microplate. OD readings were taken at 420 nm every 15 min with continuous shaking between readings for 24 h.

### Experimental Evolution

Experimental evolution was carried out using a serial transfer regime in DM25 medium for 2000 generations (~6.6 generations per day) as described previously (Elena and Lenski 2003). Relative fitness change was measured by competing evolved strains or populations against the ancestral genotype, REL606 or REL607, every 500 generations using a standard competition assay protocol. Relative fitness was defined as the ratio of the Malthusian parameter of one competitor to the other. The Malthusian parameter was calculated as follows: ${\text{m = }}{{\left( {{\text{cdx}} \times {{\text{f}}^ \wedge }{\text{x}}} \right)} \mathord{\left/ {\vphantom {{\left( {{\text{cdx}} \times {{\text{f}}^ \wedge }{\text{x}}} \right)} {{\text{cd}}0}}} \right. \kern-\nulldelimiterspace} {{\text{cd}}0}}$, where cd0 is the count of the competitor on day 0, cdx is the count of the competitor on day x, and *f* is the growth of the population over time (*x*−0). In our competitions, *f* = 100 because our transfers involve 100-fold dilution and subsequent outgrowth.

### Whole Genome Sequencing

Sequencing libraries of clones of interest were prepared by isolating 3 mg of genomic DNA from bacteria grown in 10 mL LB overnight; the isolated DNA fragmented and tagged with specific Illumina adapters using Nextera DNA sample preparation kit. The product was purified using the Zymo DNA Clean and Concentrator Kit, the libraries were dual-indexed with TruSeq Dual Indexed Sequencing primer sets, and it was ensured using an Agilent 2100 BioAnalyzer that the products were pure. Sets of compatible barcodes (11-plex) were combined into a single lane in an Illumina HiSeq 2500 rapid run flow cell (v1) after QC. Sequencing was in performed a paired end 2 × 100 base pair format (PE100) using TruSeq Rapid SBS reagents. Mutations were identified using the Breseq (0.23) pipeline (Deatherage and Barrick 2014).

Whole genome sequencing was completed for 2000 generations for eight lineages harboring ancient EF-Tu, as well as the wild-type strains. To prepare the sequencing library, we isolated 3 mg of genomic DNA from bacteria grown in 10 mL LB overnight, and fragmented and tagged the isolated DNA with specific Illumina adapters using Nextera DNA sample preparation kit. We purified the product using Zymo DNA Clean and Concentrator Kit, dual-indexed the libraries with TruSeq Dual Indexed Sequencing primer sets, and ensured that the products were pure using an Agilent 2100 BioAnalyzer. We combined the sets of compatible barcodes (11-plex) into a single lane on Illumina HiSeq 2500 Rapid Run flow cell (v1) after QC. Sequencing was performed in a paired end 2 × 100 bp format (PE100) using TruSeq Rapid SBS reagents. The Breseq (0.23) software was used for the generation and the analysis of the mutations (Deatherage and Barrick 2014).

### Fitness Measurement of the Ancestral Strain in the Presence of Over-Expressed EF-Tu

Ancient EF-Tu was cloned into a pASK-IBA43 (IBA Life Sciences) vector inducible under a tetracycline promoter using the primers (Forward) 5′ GTTGGAATTCATGTCTAAAGAAAAGTTTGAACGTAC 3′ and (Reverse) 5′ CGGGATCCTCAAGCGATGATTTTCGCAACCAC 3′, between the *Xho* and *Nde* sites. Ligation was confirmed using Forward primer 5′ GAGTTATTTTACCACTCCCT 3′ and Reverse primer 5′ CGCAGTAGCGGTAAACG 3′. The plasmid was transformed into REL606 Δ*tufA*, Δ*tufB:AncientEFTu* cells via electroporation, and transformants selected on LB agar plate with chloramphenicol. Five representative colonies were picked and preconditioned in LB media containing 250 µM anhydrous tetracycline for 24 h, followed by a 1:100 dilution into DM media containing glucose. Over-expression of the EF-Tu protein was confirmed through SDS-PAGE analysis in comparison to ancestral cells that harbored no plasmid or non-induced plasmid. A REL607 strain was acclimated to the competition environment by separate growth under the same environmental conditions as REL606 Δ*tufA*, Δ*tufB:AnEF* harboring pASK-IBA43 with the ancient EF-Tu gene. The competitors were then mixed in 50:50 ratios by volume by diluting each into fresh DM25 supplemented with 250 µM anhydrous tetracycline. Samples were plated on tetrazolium arabinose agar plate every 4 h during the 24-h competition. The competitions were carried out twice to increase the precision of fitness estimates.

### Luciferase Assay

#### tufB and pBBRlux Plasmid Cloning

The wild-type and mutant (evolved) promoter region of *tufB* gene (EF-Tu protein) was cloned into the pBBRlux plasmid as adapted from (Lenz et al. 2004) (kindly provided by Prof. Brian Hammer, Georgia Tech). Phusion High-Fidelity DNA polymerase, dNTPs, restriction enzymes (high fidelity), and T4 ligases were all obtained from New England Biolabs. DNA purification materials were purchased from QIAGEN. Promoters were amplified using PCR primers 5′-CAGAATGAAAATCAGGTAGCCGAGTTCCAG-3′ and 5′-TAGTGATTGCAGCGGTCAGCGTTGTTTTAC-3′ and resulted in a 403-bp product from REL606 *E. coli* in the 4155251–4155654 region of the genome. Restriction sites were subsequently added to the ends of the *tufB* promoter with the following primers: Forward 5′-GATACTAGTCAGAATGAAAATCAGGTAGCCGAGTTCCAG-3′ and Reverse 5′-TATGGATCCTAGTGATTGCAGCGGTCAGCGTTGTTTTAC-3′ (the underlying restriction sites correspond to *Spe*I and *Bam*HI, respectively). The EF-Tu promoter was cloned upstream of the luciferase operon in the pBBRlux plasmid in order to drive transcription. pBBRlux provides chloramphenicol (CMP) resistance.

#### Scintillation Counts

Four experimental constructs: +86/−29 (G+86A), +54/−61 (G+54T), +87/−28 (A+87 C), and +94/−21 (19-bp duplication, +96), and two control constructs: P (no promoter) and Patuf (wild-type, or unevolved ancestor, *tufB* promoter) were transformed into chemically competent *E. coli* (REL606) cells and incubated at 37 °C for 24 h on chloramphenicol (CMP) agar plates. A single colony was cultured in LB media containing CMP at 37 °C for 24 h. A 100-µL aliquot of the overnight culture was diluted one thousand-fold prior to being transferred into a 50-mL Erlenmeyer flask containing 9.9 mL of DM25 media. Cells were grown for ~8.25 h, or ~5 doublings as monitored by plating (this represents the end of log growth since these cultures reach stationary phase after ~6.6 generations in DM25) and then pelleted. The supernatant was aspirated until 100 µL of media remained, and the pellet was then resuspended in the remaining 100 µL supernatant. Scintillation counting was used to quantify the amount of light signal generated by the luciferase pathway. For all six constructs, three readings per sample were averaged for each of the two replicates assayed.

### Bacterial Enumeration

For each construct, a 10-µL aliquot was serially diluted 50 thousand-fold and 50 µL was plated on agar petri dishes containing CMP. Extrapolation was utilized to determine the total amount of cells in each scintillation assay. Three plates per flask were averaged.

#### Luciferase Assay Statistical Analysis

The luciferase expression per cell was normalized by

$$\frac{{{\rm{Total~scintillation~counts}}}}{{{\rm{Total~number~of~cells}}}}.$$

Luciferase expression for each construct was subtracted by the amount of luciferase signal from P to eliminate any leaky expression from the pBBRlux vector without promoter and presented as fold change relative to the amount of luciferase signal from Patuf. A one-way ANOVA with *α* = 0.05 and a post hoc Tukey’s HSD test were performed against Patuf to determine significant differences.

### Protein Biochemistry

#### Cloning, Expression, and Purification of Modern EF-Tu and Ancient EF-Tu Proteins

Both of the EF-Tu genes were ligated into pET15b plasmid between BamH1/EcoR1 sites, containing an N-term His-Tag with Ampicillin resistance. For expression, the plasmids were transferred in a BL21(DE3) strain and the cells were grown in LB media until OD_600_ reached 0.6–0.8 and then induced with 1 mM imidazole for 4 h. The cells were lysed using BugBuster protein extraction reagent (EMD Millipore) containing benzonase. For purification of the His-tagged protein from the supernatant, the cleared lysate was transferred into nitrilotriacetic acid (Ni-NTA) resin gravity-flow columns (Qiagen, Hilden, Germany) at 4 °C that was pre-equilibrated with lysis buffer (50 mM NaH_2_PO_4_, 300 mM NaCl, 10 mM imidazole, pH 8). The Ni-NTA gravity-flow column was washed two times with lysis buffer containing 20 mM imidazole. His-tagged protein was eluted using elution buffer (50 mM NaH_2_PO_4_, 300 mM NaCl, 200 mM imidazole, pH 8).

#### Cloning, Expression, and Purification of NusA Proteins

Both the wild-type *nusA* and the evolved *nusA* genes were amplified from their host bacterial genome using Forward primer 5′-GTGAAGGTGTCGACGCTGCGTGCGCT-3′ and Reverse primer 5′-AGCGCACGCAGCGTCGACACCTTCAC-3′. The amplified DNA was purified through gel extraction, removed from salt, and then cloned into a pET15b vector (Novagen) using 5′ GGCGACATATGAACAAAGAAATTTTGGC 3′ and 5′ GGAGCTCGAGTTACGCTTCGTCACCGA 3′ primers in between BamH1 and XhoI sites. The plasmids were transferred into a BL21(DE3) strain for expression and induced by IPTG. Cells were broken by French Press in Buffer A (20 mM Tris–HCl at pH 7.5, 50 mM MgCl_2_, 200 mM NaCl, 5% glycerol). 25 μM GDP was added in Buffer A for EF-Tu purification. After centrifugation for 30 min at 16,000 rpm (F21-8 × 50 rotor, Thermo), the supernatant was applied to Ni-NTA column and eluted with gradient Buffer B (Buffer A supplied with 500 mM imidazole). To prepare EF-Tu and NusA for ITC experiment, the proteins were dialyzed in Buffer C (20 mM Tris–HCl at pH 7.5, 50 mM MgCl_2_, 100 mM KCl) for 16 h at 4 °C.

#### ITC Analysis

The ITC data were measured on a Microcal ITC200 System (GE Healthcare). The syringe was loaded with 42 μL of 0.6-1 mM NusA and the sample cell was filled with 10 μM EF-Tu. NusA was titrated (2.5 μL for each) into EF-Tu with 120-s intervals and the first injection was 0.25 μL. The stirring speed was set at 1000 rpm. Blank experiment was performed by titrating NusA into Buffer C (20 mM Tris–HCl at pH 7.5, 50 mM MgCl_2_, 100 mM KCl).

### LC–MS/MS Analysis

#### Sample Preparation

Whole cell lysate was generated from each of the ancestral and evolved strains using Bug Buster reagent (EMD Millipore), following manufacturer’s instructions. Total protein was quantified via BCA assay using Pierce BCA protein assay kit (Thermo Fisher Scientific). 30 mg of whole cell lysate was submitted to the Proteomics and Metabolomics Facility at Colorado State University. Samples were processed for in-solution trypsin digestion as previously described (Schauer et al. 2013). Briefly, protein was precipitated out of solution in the presence of 4 volumes of 100% −20 °C acetone and then resolubilized in 8 M urea and 0.2% ProteaseMAX^TM^ surfactant trypsin enhancer (Promega, Madison, WI). Samples were reduced and alkylated with 5 mM dithiothreitol and 5 mM iodoacetamide. Trypsin (MS Grade, Thermo Pierce, San Jose, CA) was added at an enzyme-to-substrate ratio of 1:50 and incubated at 37 °C for 3-h. Trypsin was deactivated with the addition of 5% trifluoroacetic acid and desalted using C18 OMIX tips (Agilent Technologies, Santa Clara, CA) using manufacturer’s instructions. Peptide eluate was dried in a vacuum evaporator and resuspended in 3% acetonitrile/0.1% formic acid at a concentration of approximately 1 µg/µL. Relative Quantitation of EF-Tu proteins was carried out using spectral counting approach. Approximately 2 µg of tryptic digest for each sample was injected using an EASY nanoLC-II system (Thermo Scientific, San Jose, CA). Peptides were purified and concentrated using an online enrichment column (EASY-Column, 100 µm ID × 2 cm ReproSil-Pur C18). Subsequent chromatographic separation was performed on a reverse phase nanospray column (EASY-Column, 3 µm, 75 µm ID × 100 mm ReproSil-Pur C18) using a 180 min linear gradient from 10 to 55% buffer B (100% ACN, 0.1% formic acid) at a flow rate of 400 nanoliters/min. Peptides were eluted directly into the mass spectrometer (Thermo Scientific Orbitrap Velos). The instrument was operated in Orbitrap-LTQ mode where precursor measurements were acquired in the Orbitrap (60,000 resolution) and MS/MS spectra (top 20) were acquired in the LTQ ion trap with normalized collision energy of 35%. Mass spectra were collected over an m/z range of 400–2000 Da using a dynamic exclusion limit of 2 MS/MS spectra of a given peptide mass for 30 s (exclusion duration of 90 s). Compound lists of the resulting spectra were generated using Xcalibur 2.2 software (Thermo Scientific) with a S/N threshold of 1.5 and 1 scan/group.

#### Data Analysis: Spectral Counting

*Database searching* Tandem mass spectra were extracted, charge state deconvoluted, and deisotoped by ProteoWizard version 3.0. All MS/MS samples were analyzed using Mascot (Matrix Science, London, UK; version 2.3.02). Mascot was set up to search the Uniprot_e_coli_custom_reverse database (Updated August 2014, 8750 entries) (Elias and Gygi 2010) assuming the digestion enzyme trypsin, allowing up to 3 missed cleavages. Mascot was searched with a fragment ion mass tolerance of 0.80 Da and a parent ion tolerance of 20 PPM. Oxidation of methionine M (+15.99) and carbamidomethyl of cysteine C (+57) were specified in Mascot as variable modifications.

#### Criteria for Protein Identification

Scaffold (version Scaffold_4.3.4, Proteome Software Inc., Portland, OR) was used to validate MS/MS-based peptide and protein identifications. Peptide identifications were accepted if they could be established at greater than 69.0% probability to achieve an FDR less than 0.1% by the Scaffold Local FDR algorithm. Protein identifications were accepted if they could be established at greater than 99.0% probability to achieve an FDR less than 1.0% and contained at least 2 identified peptides (Kall et al. 2008; Keller et al. 2002). Protein probabilities were assigned by the Protein Prophet algorithm (Nesvizhskii et al. 2003). Proteins that contained similar peptides and could not be differentiated based on MS/MS analysis alone were grouped to satisfy the principles of parsimony.

#### Quantitative Analysis

Binary comparisons were created in separate Scaffold files comparing wild-type *E. coli* REL606 and unevolved ancestor harboring the ancient protein and the evolved lineages tested (biological replicates *n* = 3) to strain/treatment group (each *n* = 3). Biological samples were organized into categories based on strain type. Each category had 3 biological replicates. Normalization of spectral counts was not applied based on these criteria: An equal amount of sample from each replicate was loaded into the mass spectrometer and there was no deviation in processing and the number of spectra between samples is closely similar (% CV < 5% between biological replicates). Spectral counting uses the sum of the MS/MS spectra assigned to each protein as a measure of abundance (Paoletti and Washburn 2006). A *t* test was performed on the total spectral count for each MS sample using the embedded algorithm in Scaffold v 4.3.4. Proteins with P values less than 0.05 are excluded in the calculation of fold changes compared to *E. coli* REL606.

### Reconstruction of the Ancestral nusA Protein

Bacterial nusA sequences were retrieved from GenBank database. Phylogenetic tree was constructed with MrBayes (Altekar et al. 2004, Ronquist, 2003). Ancestral sequences were calculated with PAML (Yang 2007). Ancestral EF-Tu sequence was obtained from the study of Gaucher et al. (2008).

## Electronic supplementary material

Below is the link to the electronic supplementary material.

Supplementary material 1 (TIFF 22343 KB)

Supplementary material 2 (TIFF 17749 KB)

Supplementary material 3 (TIFF 22855 KB)

Supplementary material 4 (TIFF 38132 KB)

Supplementary material 5 (TIFF 10098 KB)

Supplementary material 6 (XLSX 16 KB)

Supplementary material 7 (DOCX 16 KB)

## References

[CR1] Abdulkarim F, Hughes D (1996). Homologous recombination between the tuf genes of Salmonella typhimurium. J Mol Biol.

[CR2] Acevedo-Rocha CG, Fang G, Schmidt M, Ussery DW, Danchin A (2013). From essential to persistent genes: a functional approach to constructing synthetic life. Trends Genet.

[CR3] Agashe D, Martinez-Gomez NC, Drummond DA, Marx CJ (2013). Good codons, bad transcript: large reductions in gene expression and fitness arising from synonymous mutations in a key enzyme. Mol Biol Evol.

[CR4] Agirrezabala X, Frank J (2009). Elongation in translation as a dynamic interaction among the ribosome, tRNA, and elongation factors EF-G and EF-Tu. Q Rev Biophys.

[CR5] Altekar G, Dwarkadas S, Huelsenbeck JP, Ronquist F (2004). Parallel Metropolis coupled Markov chain Monte Carlo for bayesian phylogenetic inference. Bioinformatics.

[CR6] Andersson DI, Hughes D (2009). Gene amplification and adaptive evolution in bacteria. Annu Rev Genet.

[CR7] Barrick JE, Lenski RE (2013). Genome dynamics during experimental evolution. Nat Rev Genet.

[CR001] Barrick JE, Yu DS, Yoon SH, Jeong H, Oh TK, Schneider D, Lenski RE, Kim JF (2009). Genome evolution and adaptation in a long-term experiment with *Escherichia coli*. Nature.

[CR8] Bar-Rogovsky H, Stern A, Penn O, Kobl I, Pupko T, Tawfik DS (2015). Assessing the prediction fidelity of ancestral reconstruction by a library approach. Protein Eng Des Sel.

[CR9] Battistuzzi FU, Feijao A, Hedges SB (2004). A genomic timescale of prokaryote evolution: insights into the origin of methanogenesis, phototrophy, and the colonization of land. BMC Evol Biol.

[CR10] Bell G (2016) Experimental macroevolution. Proc Biol Sci 283 10.1098/rspb.2015.2547 PMC4721102 26763705

[CR11] Benner SA (1995) Reconstructing ancient forms of life. J Cell Biochem: 200

[CR12] Bershtein S, Serohijos AW, Bhattacharyya S, Manhart M, Choi JM, Mu W, Zhou J, Shakhnovich EI (2015). Protein homeostasis imposes a barrier on functional integration of horizontally transferred genes in bacteria. PLoS Genet.

[CR13] Biasini M, Bienert S, Waterhouse A, Arnold K, Studer G, Schmidt T, Kiefer F, Cassarino TG, Bertoni M, Bordoli L, Schwede T (2014). SWISS-MODEL: modelling protein tertiary and quaternary structure using evolutionary information. Nucleic Acids Res.

[CR14] Blattler CL, Higgins JA (2014). Calcium isotopes in evaporites record variations in Phanerozoic seawater SO_4_ and Ca. Geology.

[CR15] Blount ZD (2015). The unexhausted potential of *E. coli*. Elife.

[CR16] Brandis G, Bergman JM, Hughes D (2016). Autoregulation of the tufB operon in Salmonella. Mol Microbiol.

[CR17] Bridgham JT, Ortlund EA, Thornton JW (2009). An epistatic ratchet constrains the direction of glucocorticoid receptor evolution. Nature.

[CR18] Caldas TD, El Yaagoubi A, Richarme G (1998). Chaperone properties of bacterial elongation factor EF-Tu. J Biol Chem.

[CR19] Caldas T, Laalami S, Richarme G (2000). Chaperone properties of bacterial elongation factor EF-G and initiation factor IF2. J Biol Chem.

[CR20] Chang BS, Jonsson K, Kazmi MA, Donoghue MJ, Sakmar TP (2002). Recreating a functional ancestral archosaur visual pigment. Mol Biol Evol.

[CR21] Charlebois RL, Doolittle WF (2004). Computing prokaryotic gene ubiquity: Rescuing the core from extinction. Genome Res.

[CR002] Conrad TM, Joyce AR, Applebee MK, Barrett CL, Xie B, Gao Y, Palsson BO (2009). Whole-genome resequencing of *Escherichia coli* K-12 MG1655 undergoing short-term laboratory evolution in lactate minimal media reveals flexible selection of adaptive mutations. Genome Biol.

[CR22] Conrad TM, Lewis NE, Palsson BO (2011). Microbial laboratory evolution in the era of genome-scale science. Mol Syst Biol.

[CR23] Copley SD (2003). Enzymes with extra talents: moonlighting functions and catalytic promiscuity. Curr Opin Chem Biol.

[CR24] Copley SD (2012). Toward a systems biology perspective on enzyme evolution. J Biol Chem.

[CR25] Coulomb S, Bauer M, Bernard D, Marsolier-Kergoat MC (2005). Gene essentiality and the topology of protein interaction networks. Proc Biol Sci.

[CR26] Counago R, Chen S, Shamoo Y (2006). In vivo molecular evolution reveals biophysical origins of organismal fitness. Mol Cell.

[CR27] Czworkowski J, Moore PB (1996). The elongation phase of protein synthesis. Prog Nucleic Acid Res Mol Biol.

[CR28] Datsenko KA, Wanner BL (2000). One-step inactivation of chromosomal genes in *Escherichia coli* K-12 using PCR products. Proc Natl Acad Sci USA.

[CR29] Dean AM, Thornton JW (2007). Mechanistic approaches to the study of evolution: the functional synthesis. Nat Rev Genet.

[CR30] Deatherage DE, Barrick JE (2014). Identification of mutations in laboratory-evolved microbes from next-generation sequencing data using bresEq. Methods Mol Biol.

[CR003] Dillon MM, Rouillard NP, Van Dam B, Gallet R, Cooper VS (2016). Diverse phenotypic and genetic responses to short-term selection in evolving *Escherichia coli* populations. Evolution.

[CR31] Dragosits M, Mattanovich D (2013). Adaptive laboratory evolution—principles and applications for biotechnology. Microb Cell Factor.

[CR32] Drummond DA, Bloom JD, Adami C, Wilke CO, Arnold FH (2005). Why highly expressed proteins evolve slowly. Proc Natl Acad Sci USA.

[CR33] Elena SF, Lenski RE (2003). Evolution experiments with microorganisms: The dynamics and genetic bases of adaptation. Nat Rev Genet.

[CR34] Elias JE, Gygi SP (2010). Target-decoy search strategy for mass spectrometry-based proteomics. Methods Mol Biol.

[CR35] Fischer N, Neumann P, Konevega AL, Bock LV, Ficner R, Rodnina MV, Stark H (2015). Structure of the *E. coli* ribosome-EF-Tu complex at <3 angstrom resolution by C-s-corrected cryo-EM. Nature.

[CR36] Gaucher EA, Thomson JM, Burgan MF, Benner SA (2003). Inferring the palaeoenvironment of ancient bacteria on the basis of resurrected proteins. Nature.

[CR37] Gaucher EA, Govindarajan S, Ganesh OK (2008). Palaeotemperature trend for Precambrian life inferred from resurrected proteins. Nature.

[CR38] Goldman AD, Samudrala R, Baross JA (2010). The evolution and functional repertoire of translation proteins following the origin of life. Biol Direct.

[CR39] Gong LI, Bloom JD (2014). Epistatically interacting substitutions are enriched during adaptive protein evolution. PLoS Genet.

[CR40] Gould SJ (1989). Wonderful life: the burgess shale and the nature of history.

[CR41] Gromiha MM, Oobatake M, Sarai A (1999). Important amino acid properties for enhanced thermostability from mesophilic to thermophilic proteins. Biophys Chem.

[CR42] Harms MJ, Thornton JW (2013). Evolutionary biochemistry: revealing the historical and physical causes of protein properties. Nat Rev Genet.

[CR43] Herring CD, Glasner JD, Blattner FR (2003). Gene replacement without selection: regulated suppression of amber mutations in Escherichia coli. Gene.

[CR44] Herron MD, Doebeli M (2013). Parallel evolutionary dynamics of adaptive diversification in Escherichia coli. PLoS Biol.

[CR45] Hobbs JK, Prentice EJ, Groussin M, Arcus VL (2015). Reconstructed ancestral enzymes impose a fitness cost upon modern bacteria despite exhibiting favourable biochemical properties. J Mol Evol.

[CR46] Hoekstra HE, Coyne JA (2007). The locus of evolution: evo devo and the genetics of adaptation. Evolution Int J org Evolution.

[CR47] Huelsenbeck JP, Bollback JP (2001). Empirical and hierarchical Bayesian estimation of ancestral states. Syst Biol.

[CR48] Ito K, Uno M, Nakamura Y (1998). Single amino acid substitution in prokaryote polypeptide release factor 2 permits it to terminate translation at all three stop codons. Proc Natl Acad Sci USA.

[CR49] Jacob F, Monod J (1961). Genetic regulatory mechanisms in the synthesis of proteins. J Mol Biol.

[CR50] Jain R, Rivera MC, Lake JA (1999). Horizontal gene transfer among genomes: the complexity hypothesis. Proc Natl Acad Sci USA.

[CR51] Kacar B, Ramsey G, Pence C (2016). Rolling the dice twice: evolving reconstructed ancient proteins in extant organisms. Chance in evolution.

[CR52] Kacar B, Gaucher EA (2012) Towards the Recapitulation of Ancient History in the Laboratory: Combining Synthetic Biology with Experimental Evolution Proceedings of the Thirteenth International Conference on the Simulation and Synthesis of Living Systems. MIT Press: 11–18

[CR53] Kacar B, Gaucher EA (2013). Experimental evolution of protein-protein interaction networks. Biochem J.

[CR54] Kacar B, Adam ZR, Hanson-Smith V, Boekelheide N (2016) Constraining the Great Oxidation Event within the Rubisco phylogenetic tree The Geological Society of America, p 289 10.1111/gbi.12243 PMC5575542 28670785

[CR55] Kall L, Storey JD, MacCoss MJ, Noble WS (2008). Assigning significance to peptides identified by tandem mass spectrometry using decoy databases. J Proteome Res.

[CR56] Kavaliauskas D, Nissen P, Knudsen CR (2012). The busiest of all ribosomal assistants: elongation factor Tu. BioChemistry.

[CR57] Keller A, Nesvizhskii AI, Kolker E, Aebersold R (2002). Empirical statistical model to estimate the accuracy of peptide identifications made by MS/MS and database search. Anal Chem.

[CR58] Knauth PL (2004). Temperature and salinity history of the Precambrian ocean: implications for the course of microbial evolution. Palaeogeogr Palaeoclimatol Palaeoecol.

[CR59] Kryazhimskiy S, Rice DP, Jerison ER, Desai MM (2014). Microbial evolution. Global epistasis makes adaptation predictable despite sequence-level stochasticity. Science.

[CR60] Kvitek DJ, Sherlock G (2011). Reciprocal sign epistasis between frequently experimentally evolved adaptive mutations causes a rugged fitness landscape. PLoS Genet.

[CR61] Lang GI, Desai MM (2014). The spectrum of adaptive mutations in experimental evolution. Genomics.

[CR62] Larios-Sanz M, Travisano M (2009). Experimental evolution of an essential Bacillus gene in an *E. coli* host. Methods Mol Biol.

[CR63] Lee JS, An G, Friesen JD, Fill NP (1981). Location of the tufB promoter of *E. coli*: cotranscription of tufB with four transfer RNA genes. Cell.

[CR006] Lenz DH, Mok KC, Lilley BN, Kulkarni RV, Wingreen NS, Bassler BL (2004). The small RNA chaperone Hfq and multiple small RNAs control quorum sensing in *Vibrio harveyi* and *Vibrio cholerae*. Cell.

[CR64] Liberles DA (2007). Using phylogeny to understand genomic evolution Parsimony, Phylogeny, and Genomics.

[CR65] Lind PA, Andersson DI (2013). Fitness costs of synonymous mutations in the rpsT gene can be compensated by restoring mRNA base pairing. PLoS ONE.

[CR66] Lind PA, Tobin C, Berg OG, Kurland CG, Andersson DI (2010). Compensatory gene amplification restores fitness after inter-species gene replacements. Mol Microbiol.

[CR68] Lunzer M, Milter SP, Felsheim R, Dean AM (2005). The biochemical architecture of an ancient adaptive landscape. Science.

[CR69] Lunzer M, Golding GB, Dean AM (2010). Pervasive cryptic epistasis in molecular evolution. PLoS Genet.

[CR70] Lynch VJ, Wagner GP (2008). Resurrecting the role of transcription factor change in developmental evolution. Evolution Int J org Evolution.

[CR005] Maddamsetti R, Lenski RE, Barrick JE (2016). Adaptation, clonal interference, and frequency-dependent interactions in a long-term evolution experiment with *Escherichia coli*. Genetics.

[CR71] Miller C, Davlieva M, Wilson C, White KI, Counago R, Wu G, Myers JC, Wittung-Stafshede P, Shamoo Y (2010). Experimental evolution of adenylate kinase reveals contrasting strategies toward protein thermostability. Biophys J.

[CR72] Negrutskii BS, El’skaya AV (1998). Eukaryotic translation elongation factor 1 alpha: structure, expression, functions, and possible role in aminoacyl-tRNA channeling. Prog Nucleic Acid Res Mol Biol.

[CR73] Nesvizhskii AI, Keller A, Kolker E, Aebersold R (2003). A statistical model for identifying proteins by tandem mass spectrometry. Anal Chem.

[CR74] Pagel M (1999). Inferring the historical patterns of biological evolution. Nature.

[CR75] Paoletti AC, Washburn MP (2006). Quantitation in proteomic experiments utilizing mass spectrometry. Biotechnol Genet Eng Rev.

[CR76] Pauling L, Zuckerkandl E (1963). Chemical paleogenetics molecular restoration studies of extinct forms of life. Acta Chem Scand.

[CR77] Pena MI, Van Itallie E, Bennett MR, Shamoo Y (2010). Evolution of a single gene highlights the complexity underlying molecular descriptions of fitness. Chaos.

[CR78] Pereira-Leal JB, Levy ED, Teichmann SA (2006). The origins and evolution of functional modules: lessons from protein complexes. Philos Trans R Soc Lond B Biol Sci.

[CR004] Phillips KN, Castillo G, Wünsche A, Cooper TF (2016). Adaptation of *Escherichia coli* to glucose promotes evolvability in lactose. Evolution.

[CR79] Robert X, Gouet P (2014). Deciphering key features in protein structures with the new END script server. Nucleic Acids Res.

[CR80] Schauer KL, Freund DM, Prenni JE, Curthoys NP (2013) Proteomic profiling and pathway analysis of the response of rat renal proximal convoluted tubules to metabolic acidosis. Am J Physiol Renal Physiol 305:F628 10.1152/ajprenal.00210.2013 PMC3761203 23804448

[CR81] Schnell R, Abdulkarim F, Kalman M, Isaksson LA (2003). Functional EF-Tu with large C-terminal extensions in an *E-coli* strain with a precise deletion of both chromosomal tuf genes. FEBS Lett.

[CR82] Shazand K, Tucker J, Grunberg-Manago M, Rabinowitz JC, Leighton T (1993). Similar organization of the nusA-infB operon in Bacillus subtilis and *Escherichia coli*. J Bacteriol.

[CR83] Sievers F, Wilm A, Dineen D, Gibson TJ, Karplus K, Li WZ, Lopez R, McWilliam H, Remmert M, Soding J, Thompson JD, Higgins DG (2011) Fast, scalable generation of high-quality protein multiple sequence alignments using Clustal Omega. Mol Syst Biol 7 10.1038/msb.2011.75 PMC3261699 21988835

[CR84] Tenaillon O, Barrick JE, Ribeck N, Deatherage DE, Blanchard JL, Dasgupta A, Wu GC, Wielgoss S, Cruveiller S, Médigue C, Schneider D, Lenski RE (2016). Tempo and mode of 40 genome evolution in a 50,000-generation experiment. Nature.

[CR85] Thornton JW (2004). Resurrecting ancient genes: experimental analysis of extinct molecules. Nat Rev Genet.

[CR86] Tubulekas I, Hughes D (1993). Growth and translation elongation rate are sensitive to the concentration of EF-Tu. Mol Microbiol.

[CR87] Ugalde JA, Chang BS, Matz MV (2004). Evolution of coral pigments recreated. Science.

[CR88] Urbanczyk H, Furukawa T, Yamamoto Y, Dunlap PV (2012). Natural replacement of vertically inherited lux-rib genes of Photobacterium aquimaris by horizontally acquired homologues. Environ Microbiol Rep.

[CR89] Van Delft JH, Schmidt DS, Bosch L (1987). The tRNA-tufB operon transcription termination and processing upstream from tufB. J Mol Biol.

[CR90] van der Meide PH, Vijgenboom E, Talens A, Bosch L (1983). The role of EF-Tu in the expression of tufA and tufB genes. Eur J Biochem.

[CR91] Yang Z (2007). PAML 4: phylogenetic analysis by maximum likelihood. Mol Biol Evol.

[CR92] Zengel JM, Lindahl L (1982). A secondary promoter for elongation factor Tu synthesis in the str ribosomal protein operon of *Escherichia coli*. Mol Gen Genet.

[CR93] Zhou Y, Asahara H, Gaucher EA, Chong S (2012). Reconstitution of translation from Thermus thermophilus reveals a minimal set of components sufficient for protein synthesis at high temperatures and functional conservation of modern and ancient translation components. Nucleic Acids Res.

[CR94] Zhu GP, Golding GB, Dean AM (2005). The selective cause of an ancient adaptation. Science.

[CR95] Zotenko E, Mestre J, O’Leary DP, Przytycka TM (2008). Why do hubs in the yeast protein interaction network tend to be essential: reexamining the connection between the network topology and essentiality. PLoS Comput Biol.

