# Supplementary figures and images for: Experimental Evolution of Escherichia coli Harboring an Ancient Translation Protein
Source: J Mol Evol. 2017 Feb 23;84(2):69–84. doi: 10.1007/s00239-017-9781-0 (PMC5371648; doi:10.1007/s00239-017-9781-0)

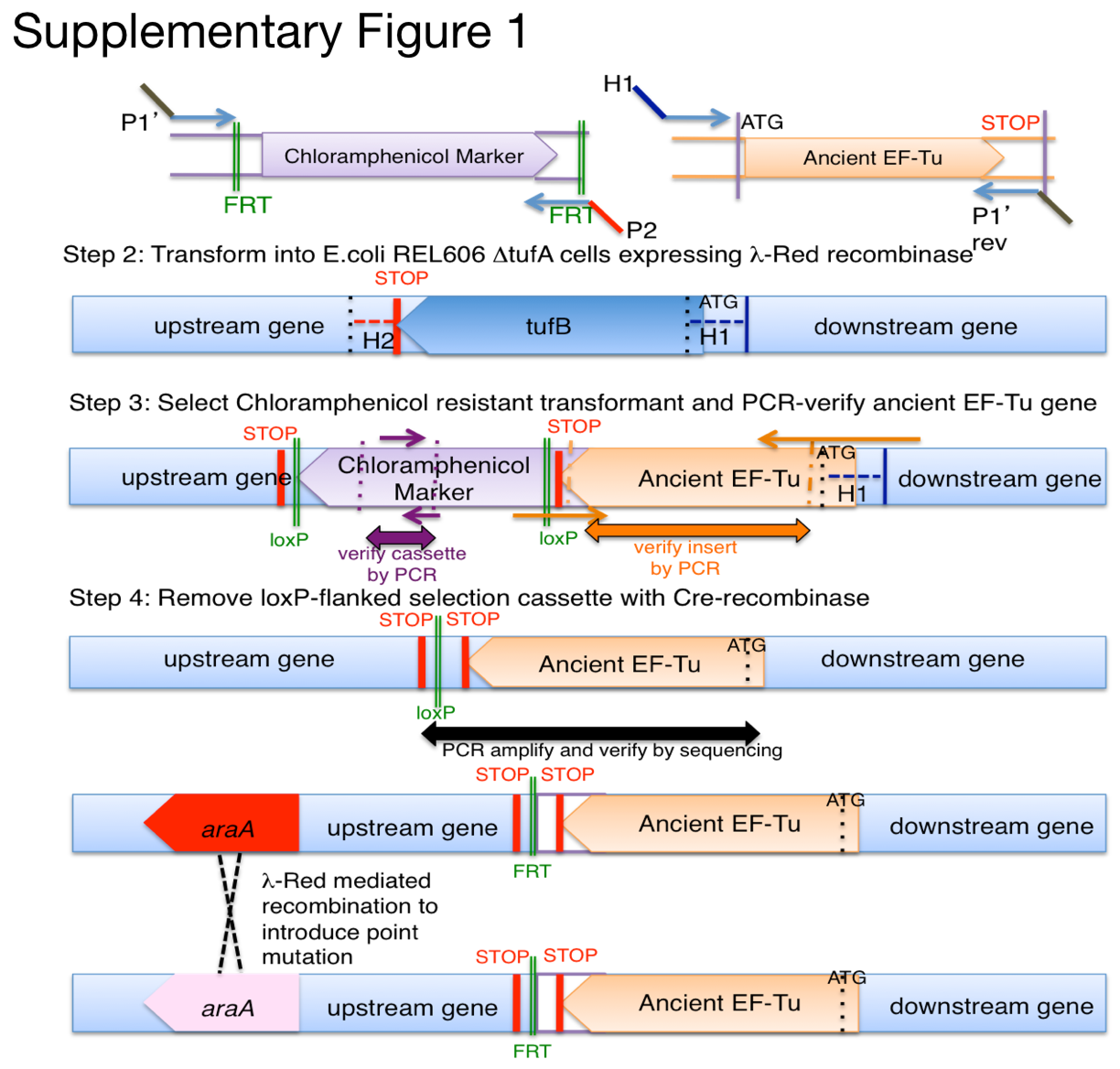

Supplement: Supplementary file 1 — Supplementary material 1 (TIFF 22343 KB) [file 239_2017_9781_MOESM1_ESM.tiff]

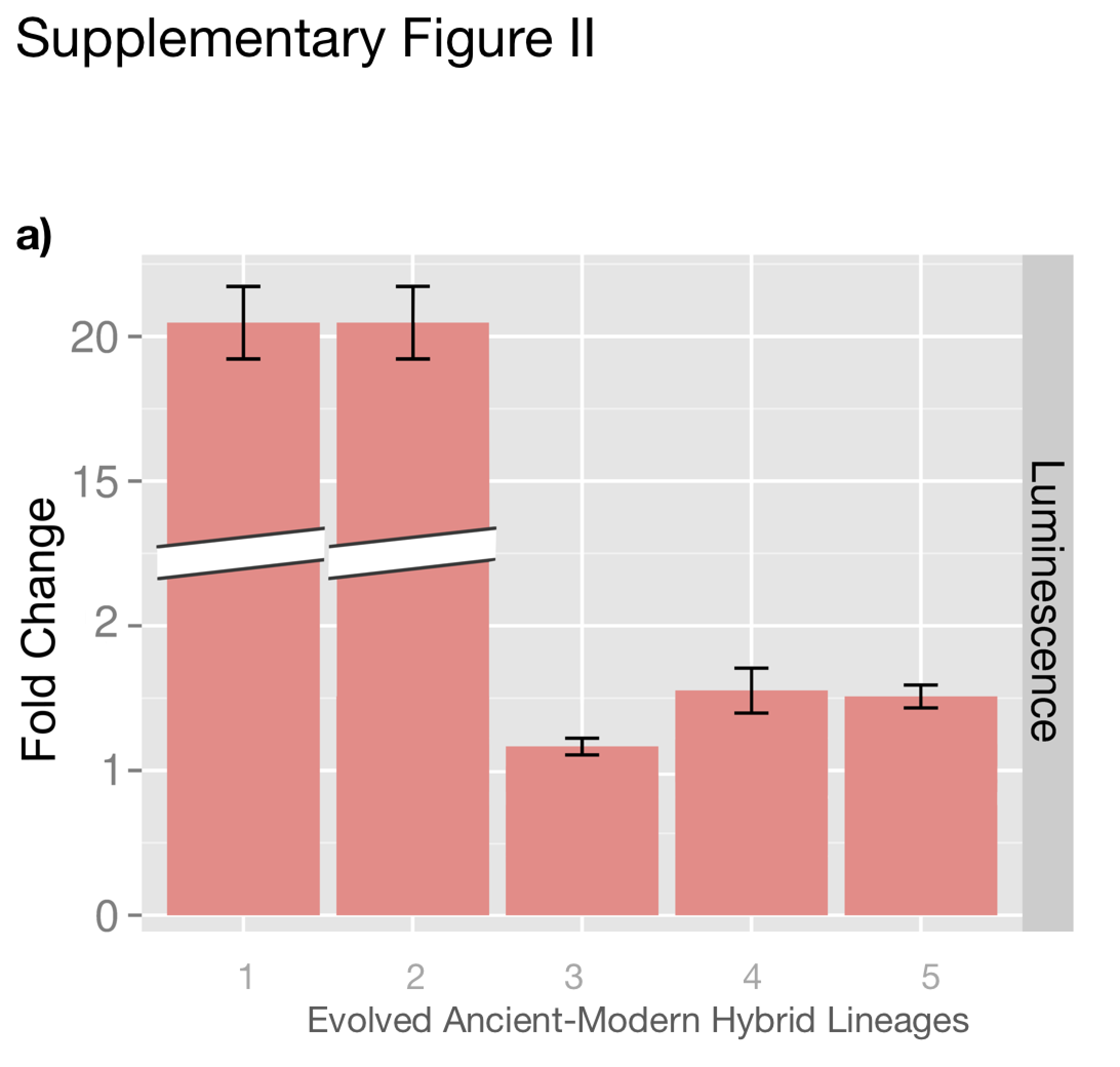

Supplement: Supplementary file 2 — Supplementary material 2 (TIFF 17749 KB) [file 239_2017_9781_MOESM2_ESM.tiff]

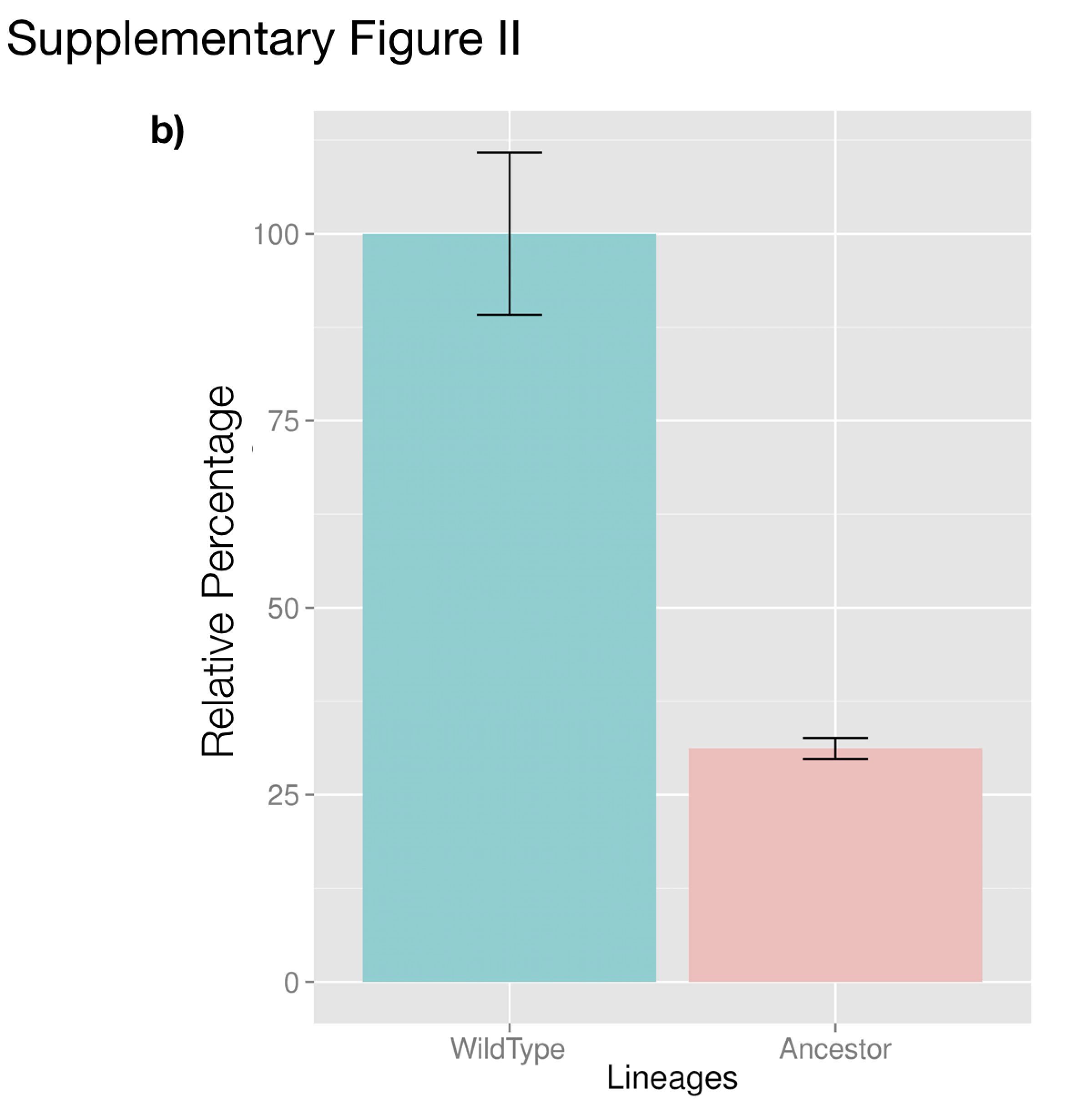

Supplement: Supplementary file 3 — Supplementary material 3 (TIFF 22855 KB) [file 239_2017_9781_MOESM3_ESM.tiff]

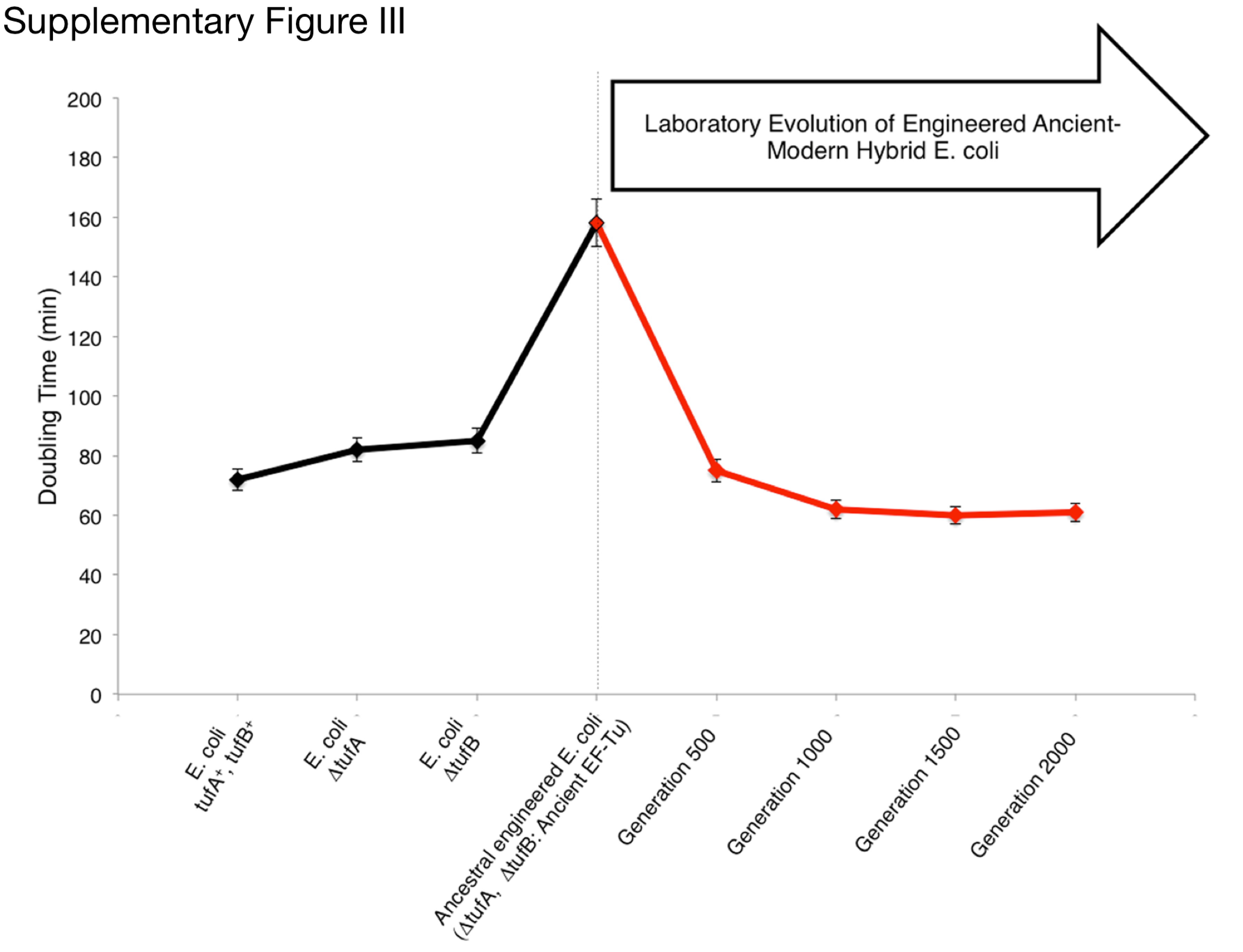

Supplement: Supplementary file 4 — Supplementary material 4 (TIFF 38132 KB) [file 239_2017_9781_MOESM4_ESM.tiff]

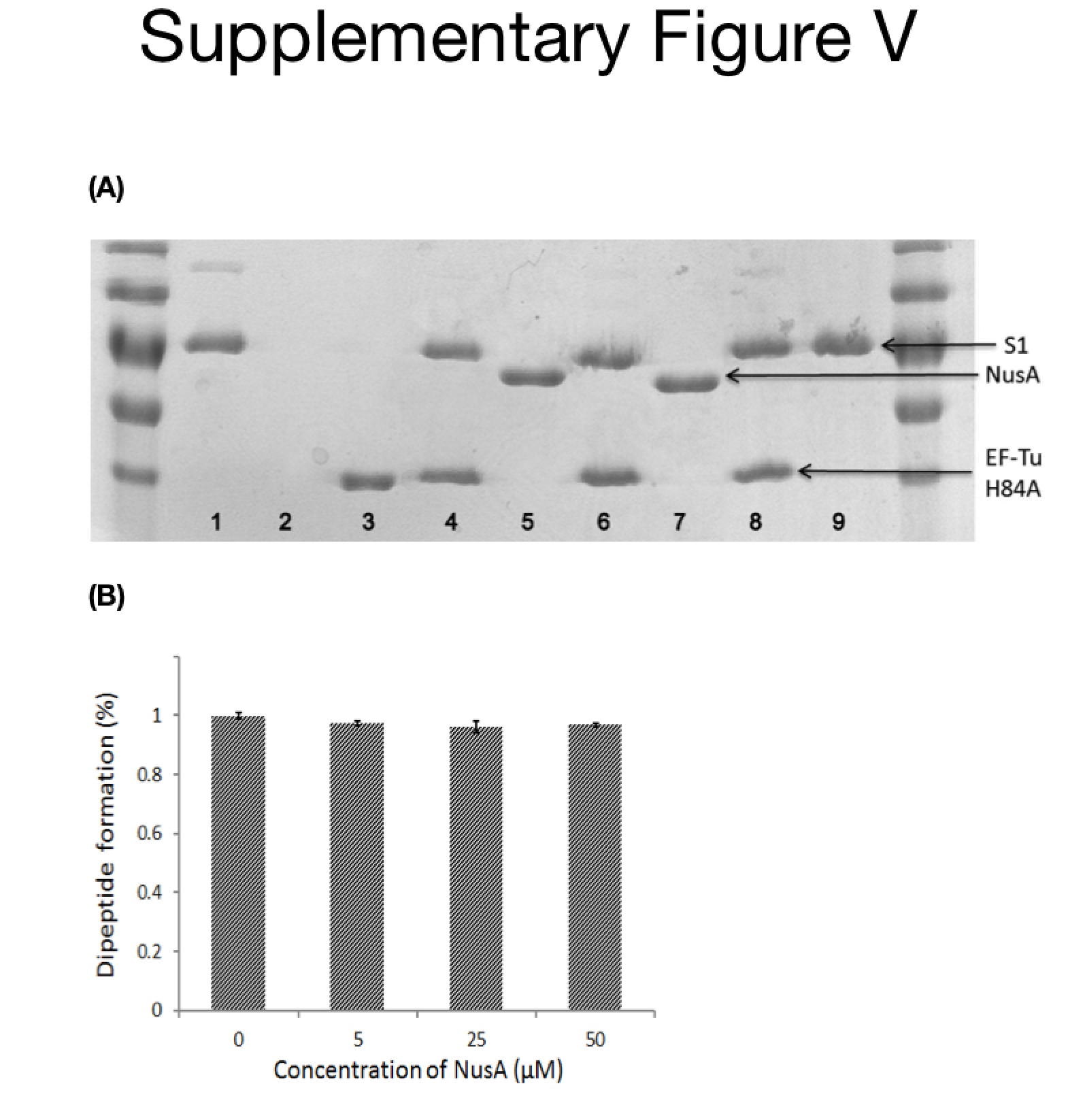

Supplement: Supplementary file 5 — Supplementary material 5 (TIFF 10098 KB) [file 239_2017_9781_MOESM5_ESM.tiff]
