# Supplementary material for: Experimental Evolution of Escherichia coli Harboring an Ancient Translation Protein
Source: J Mol Evol. 2017 Feb 23;84(2):69–84. doi: 10.1007/s00239-017-9781-0 (PMC5371648; doi:10.1007/s00239-017-9781-0)
Supplement: Supplementary file 7 — Supplementary material 7 (DOCX 16 KB) [file 239_2017_9781_MOESM7_ESM.docx]

**Supplementary Material Captions**

**Supplementary Figure I**

Recombineering outline for the allelic replacement of the tufA with the ancient EF-Tu gene

**Supplementary Figure II**

1. Effect of mutations on the expression of reporter gene for the EF-Tu promoter region measured using luciferase reporter assay. B) Relative whole cell concentration of EF-Tu (percent) based on MALDI TOF MS detection

**Supplemental Figure III**

The replacement of the modern EF-Tu with the ancient EF-Tu caused E. coli to be maladapted. As the ancient-modern hybrid populations evolve, the doubling time reduces from ~70 minutes to ~45 minutes. Each red line represents the mean doubling time calculated via three representative clones for each lineage (n=7) in minimal glucose media (DM25).

**Supplemental Figure IV A**

A) SDS PAGE analysis of the ribosomal elongation complex shows that NusA cannot bind to 70S ribosome. Initiation complex (containing 70S ribosome, initiation factors Kd (μM) Wild type NusA NusA Δ9 Wild type EF-Tu 14.6 ± 5.2 153.6 ± 5.4 Ancient EF-Tu 30.1 ± 2.6 680 ± 66 (IF1, IF2 and IF3), XR7-ML mRNA and fMet-tRNAfMet) was mixed with elongation mix (containing EF-Tu GTPase mutant H84A, EF-Ts, Leu tRNA synthetase, Leucine) in the absence and presence of NusA. After incubating the reactions for 10s the mixes were loaded directly on 37% sucrose cushion (100 ml). The samples were centrifuged at 80,000 rpm for 2 hours at 4°C. Ribosomal pellets were loaded in the SDS-PAGE. The gel was stained with Coomassie blue and only the upper part of the gel showing bands corresponding to 30S protein S1, EF-Tu H84A and NusA is displayed. EF-Tu H84A was chosen to show EF-Tu retention in the ribosome since this mutant EF-Tu is defective in release.

The lanes contained following samples:

1. 70S initiation complex (IC), 2. Elongation mix (EM), 3. EF-Tu H84A protein,

4. 70S IC + EM + NusA, 5. NusA protein alone,

6. 70S IC + EM + NusAD27CTD, 7. NusAΔ9 alone,

8. 70S IC + EM, 9. 70S ribosome

The absence of NusA bands in lanes 4 and 6 suggest the lack of binding of NusA to the 70S ribosome.

**Supplemental Figure IV B**

ITC profiles for the titration of NusA to EF-Tu. NusA protein that was purified from plasmids pLT20 (wild-type nusA) (a and c), pLT21 (mutant nusA with 27bp deletion in C-terminal, nusA ΔCTD27) (b and d) was injected to 10 μM wild type (a and b) and ancient EF-Tu (c and d), respectively. The upper panel of each figure represents the raw plots of enthalpy for each injection (μcal/s) against time (min). The corresponding bottom panels show integrated heats (closed squares) in each injection against mole ratio. The data points were fitted to a one-site model, suggesting that native EF-Tu interacts with nusA with a Kd of 14.6 ± 5.2 μM.
